# A Viral Immunity Chromosome in the Marine Picoeukaryote, *Ostreococcus tauri*

**DOI:** 10.1371/journal.ppat.1005965

**Published:** 2016-10-27

**Authors:** Sheree Yau, Claire Hemon, Evelyne Derelle, Hervé Moreau, Gwenaël Piganeau, Nigel Grimsley

**Affiliations:** Sorbonne Universités, UPMC Univ Paris 06, CNRS, Biologie Intégrative des Organismes Marins (BIOM, UMR 7232), Observatoire Océanologique, Banyuls sur Mer, France; University of California Riverside, UNITED STATES

## Abstract

Micro-algae of the genus *Ostreococcus* and related species of the order Mamiellales are globally distributed in the photic zone of world's oceans where they contribute to fixation of atmospheric carbon and production of oxygen, besides providing a primary source of nutrition in the food web. Their tiny size, simple cells, ease of culture, compact genomes and susceptibility to the most abundant large DNA viruses in the sea render them attractive as models for integrative marine biology. In culture, spontaneous resistance to viruses occurs frequently. Here, we show that virus-producing resistant cell lines arise in many independent cell lines during lytic infections, but over two years, more and more of these lines stop producing viruses. We observed sweeping over-expression of all genes in more than half of chromosome 19 in resistant lines, and karyotypic analyses showed physical rearrangements of this chromosome. Chromosome 19 has an unusual genetic structure whose equivalent is found in all of the sequenced genomes in this ecologically important group of green algae.

## Author Summary

We propose that chromosome 19 of *O*. *tauri* is specialized in defence against viral attack, a constant threat for all planktonic life, and that the most likely cause of resistance is the over-expression of numerous predicted glycosyltransferase genes. *O*. *tauri* thus provides an amenable model for molecular analysis of genome evolution under environmental stress and for investigating glycan-mediated host-virus interactions, such as those seen in herpes, influenza, HIV, PBCV and mimivirus.

## Introduction

Eukaryotic micro-algae of the genus *Ostreococcus* and related species of the order Mamiellales are globally distributed in the photic zone of the world's oceans where they contribute to fixation of atmospheric carbon and production of oxygen, besides providing a primary source of nutrition in the food web [1–3]. Their tiny size (1–3 μm), simple cells (one chloroplast, one mitochondrion), ease of laboratory culture and extremely small genomes, several of which have been completely sequenced [4–8], render them attractive as models for marine ecology [9–11], cell biology [12] and evolution in the green lineage [4,13]. Typical features of the highly streamlined genomes of the Mamiellales include a higher GC content (48–64% [7,14], 59% for *O*. *tauri*) than higher plants (41%, [15]) and two unusual “outlier” lower GC chromosomes that can carry higher proportions of transposons and genes predicted to originate from prokaryotes (see [16] for a recent review). In the type species *O*. *tauri*, these two chromosomes are chromosome 2 (the big outlier chromosome or BOC) and chromosome 19 (the small outlier or SOC), both of which greatly vary in size between strains isolated from the environment [17].

In marine environments, unicellular organisms account for the largest biomass, but they are outnumbered by about ten to one by viruses, which can lyse host cells and thereby contribute to biogeochemical cycles by promoting the turnover of plankton populations [18–20]. While large DNA viruses infecting algae have been known for many years (see [21,22] for reviews), including viruses of *Micromonas pusilla*, a member of the Mamiellales [23], viruses of *Ostreococcus* spp. were discovered more recently. Complete genomes of many viruses infecting Mamiellales (genus *Prasinovirus*) are now available [24–27]. Given the size of an *Ostreococcus* cell (about 1 μm) prasinoviruses are huge icosahedral particles, about one eighth of the host cell diameter between faces (110 nm), carrying a repertoire of about 250 genes. Resistance to prasinoviruses arises spontaneously in cultured algal lines [28] and their complex host–strain specificity patterns [29,30] witness their involvement in the planktonic 'arms race' that assures for rapid turnover and evolution in micro-algal populations (see [31] for a review). Viruses of algae in general and prasinoviruses in particular play an important role in controlling phytoplankton populations [32–36], the effectiveness of host defence responses thus determines the fate of algal populations.

Regrowth of eukaryotic microalgae following viral lysis with large dsDNA viruses has been observed in phylogenetically diverse lineages [37,38] and such virus-resistant lines can be stable in culture [28,38,39]. In the diplont marine bloom-forming haptophyte *Emiliania huxleyii* viral infection of diploid cells led to the selection of haploid cells which were resistant to infection [40]. Cell lines of the toxic bloom-forming dinoflagellate *Heterocapsa circularisquama* became resistant to viral infection by HcRNAV after co-culture with this single-stranded RNA virus. In this case, resistance appeared to be reversible and a variable proportion of resistant cells in culture harboured viruses and produced particles [41]. Some of these authors suggest that changes in the host cell surface might occur, or that non-infectious defective particles in culture might occupy available receptor sites on host cells, but no specific molecular mechanism has been demonstrated.

In *O*. *tauri*, Thomas et al. [28] observed two kinds of virus-resistant cell lines, either lines which were resistant but chronically infected, producing viruses at a low level in culture (“resistant producers” referred to as R^P^), or cell lines devoid of viruses and immune to re-infection (“resistant non-producers”, or R^NP^). Here we aimed to unravel the molecular mechanisms underlying viral resistance using clonal lines of *O*. *tauri* RCC4221 [4] and its virus, OtV5. We produced numerous independent OtV5-resistant lines to examine host and viral gene expression in detail with RNA-Seq technology and to test whether altered gene expression could be responsible for the resistance phenotypes.

## Results

### Generation of virus-resistant *O*. *tauri*

**Fig 1:**
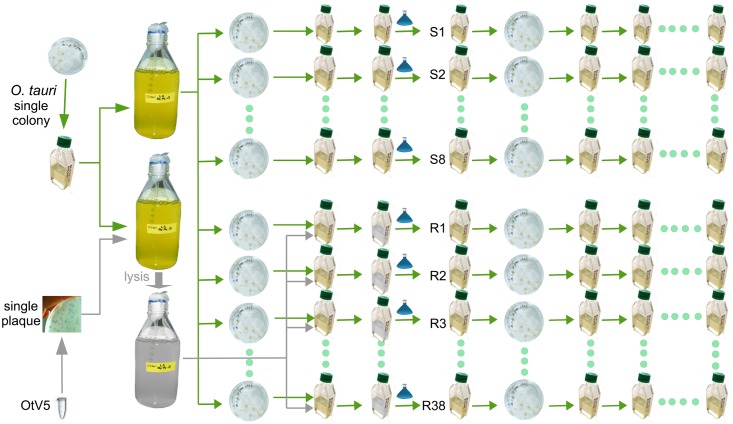
Experimental strategy for production of *Ostreococcus tauri* clonal lines susceptible or resistant to OtV5. A single colony of *O*. *tauri* was used to produce two 1-litre cultures of *O*. *tauri* cells, one used to prepare DNA for genome re-sequencing and to produce 46 independent clonal lines and another that was lysed by clonal OtV5. The viral lysate was subsequently used to inoculate 38 small flasks. Fresh medium (small blue flasks) was added to each lysate or control flask, and after ~1 week OtV5-resistant cells grew. A single colony from all lines was randomly chosen after plating and maintained in liquid culture for transcriptome sequencing and further analyses.

**Fig 2:**
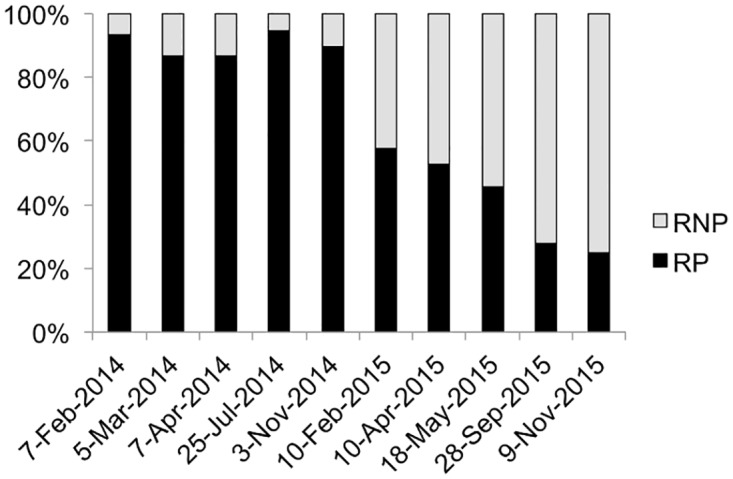
Proportion of resistant producer (RP) and resistant non-producer (RNP) lines over the course of the study. Percentage of RP and RNP in resistant lines on the ordinate and date when virus production test was performed on the abscissa.

The experimental schema in Fig 1 shows how independent clonal lines of *O*. *tauri* resistant to OtV5 were generated from a clonal starting population. The parent OtV5-susceptible *O*. *tauri* was subcultured into 46 parallel independent culture lines (one set of 30 and another set of 16). OtV5 was introduced into 38 of the *O*. *tauri* lines while eight were maintained as non-infected controls. All cultures with added OtV5 lysed, appearing clear of visible cells within 3–5 days. The cultures exposed to OtV5 showed regrowth of cells after approximately one week and, upon re-exposure to OtV5, were subsequently resistant to lysis. Periodic re-testing for OtV5 resistance showed viral resistance persisted over the almost two-year duration of the study while the control lines continued to be susceptible (8 susceptible control lines were maintained alongside the resistant lines shown in Fig 1, and at each of the 10 dates shown 8/8 lines remained susceptible to OtV5; at the end of the time course, all of the remaining 36 lines were found to be resistant). Resistant lines were periodically tested for production of infective OtV5 by taking cell-free culture medium from resistant lines, adding it to the susceptible parent and observing lysis. The majority of resistant lines were R^P^ at the start of the experiment, however, the proportion of R^P^ diminished over time so that R^NP^ lines were more abundant at the end of the study (Fig 2).

### Differentially expressed genes are clustered on chromosome 19 and mainly related to carbohydrate metabolism

Twenty-three *O*. *tauri* transcriptomes were sequenced from the experiment including 19 virus-resistant and four susceptible. To test if OtV5-resistance was linked to differential gene expression and to identify the genes involved, we compared four resistant to four susceptible transcriptomes from matching RNA library batches (S1 Table). At this early point in the study all but two lines were R^P^, so resistant lines used in the comparison were R^P^, however over the course of the study several lines of evidence indicated R^P^ and R^NP^ were probably due to similar molecular mechanisms (discussed below). Over 95% (7432 of the 7749) of *O*. *tauri* chromosomal genes analysed were transcribed to some extent (≥10 aligned reads in at least one sample) in both resistant and susceptible *O*. *tauri* lines suggesting that the majority of genes were expressed at the time of sampling.

**Fig 3:**
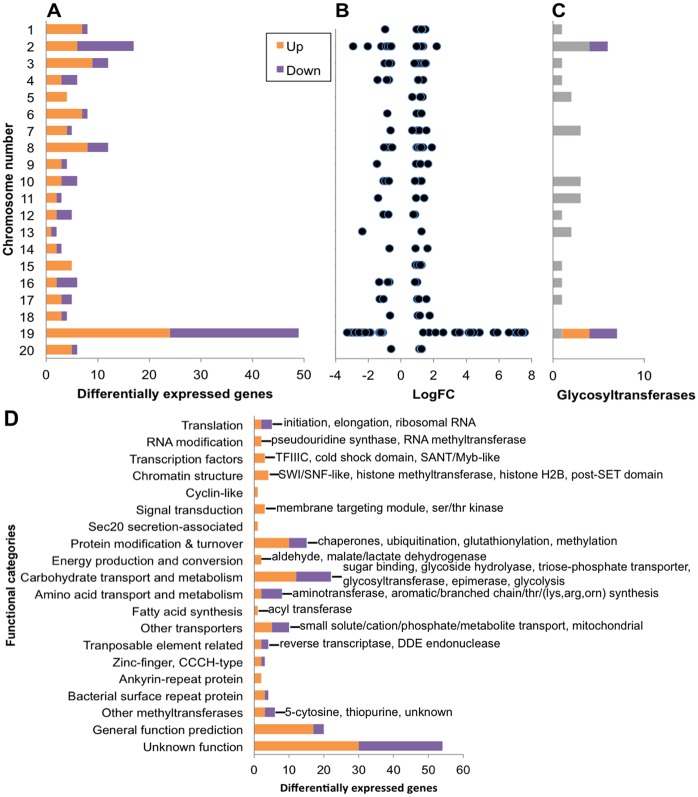
Differential gene expression in OtV5-resistant compared to susceptible control *O*. *tauri*. Up (orange) and Down (purple) regulated genes in virus resistant lines. **(A)** Counts of differentially transcribed genes per chromosome. **(B)** Plot of the log_2_ fold change values of differentially transcribed genes per chromosome. **(C)** Predicted glycosyltransferase gene counts per chromosome based on matches to InterPro domains and families associated with glycosyltransferases. Genes with no difference in regulation are shown in grey. **(D)** Counts of differentially transcribed genes grouped according to functional categories shown on the ordinate. Labels describe the genes in each category (full gene descriptions in S2 and S4 Tables). Genes located on the large inverted duplicated region on chromosome 19 were only counted once.

**Fig 4:**
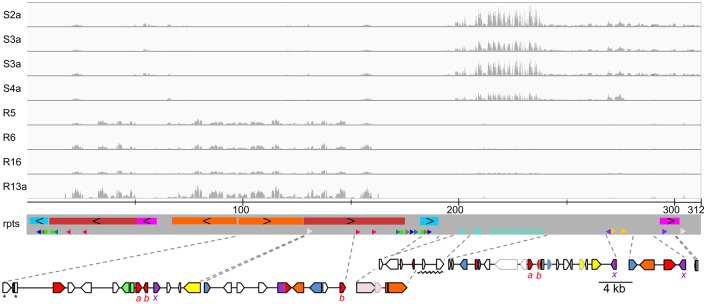
Bipartite structure of chromosome 19. Genes over-transcribed in virus-resistant lines were all located in a region of ~180 kb (on the left) containing long inverted repeats while under-transcribed genes were on a ~100 kb region (on the right). Tracks from top to bottom: alignment of RNA-Seq reads from susceptible (S2a–S5a) and resistant (R5, R6, R16 and R13a) samples (read depths have the same scale) onto chromosome 19 assembled from PacBio reads; scale in kb; (**rpts**) rectangles of the same colour on a grey background represent large duplicated blocks with black chevrons indicating inverted relative orientation and small arrowheads and chevrons of the same colour and style represent short repeats and their relative orientation; at the bottom, genomic maps zooming-in on blocks encoding genes under-transcribed **(right)** and over-transcribed **(left)** in virus-resistant lines (blue—methyltransferase, red—glycosyltransferase, green—epimerase/dehydratase, purple—sugar phosphate transporter, orange—other sugar metabolism, light pink—cell surface repeat lipoprotein, yellow—transposon-related, white—ORFan or other functions). N.B. the coloured rectangles on the grey background are **not** related to those used in the gene maps beneath. Significantly differentially transcribed genes are outlined in black (see S2 Table for full annotations of these genes) and those not differentially regulated are outlined in light grey. The gene map region underlined by a zigzag is part of a series of tandem repeats shown by light blue chevrons (see S3 Fig). *a*, *b*, *x*: Genes with the same subscript letter are putative paralogues. *These genes are duplicated as they span the ends of the adjacent inverted repeated blocks but the first gene (ostta19g00040) includes a short tract of unique intervening sequence.

A total of 170 *O*. *tauri* chromosomal genes were significantly differentially transcribed in resistant lines of which 103 were up-regulated and 67 down-regulated (Fig 3, and see S2 and S4 Tables for full gene descriptions), which represents only 2% of all expressed *O*. *tauri* genes. Most strikingly, 49 differentially transcribed genes, representing almost a third of differentially transcribed genes were concentrated on chromosome 19, the SOC (Fig 3A). These genes on chromosome 19 also had overall larger log_2_ fold change values indicating larger changes in expression levels on genes from this chromosome than the others (Fig 3B). Predicted glycosyltransferases (GTs) appear to be enriched on chromosome 19 compared to the other chromosomes, the majority of which were differentially transcribed in OtV5-resistant lines (Fig 3C). The only other chromosome encoding GTs regulated in virus resistant lines was chromosome 2, the BOC, where the GTs were down-regulated. Annotated genes on chromosome 19 are known to belong to few functional categories [5] relating to surface membrane proteins, the building of glycoconjugates, as well as methyltransferases (MTs) (Fig 4, S2 Table). The majority (16 of 22) of differentially transcribed carbohydrate transport and metabolism genes (Fig 3D) were located on chromosome 19. Furthermore, almost half of the differentially transcribed genes of unknown function (29 of 54) (Fig 3D) were also encoded by chromosome 19. Thus, genes involved in virus immunity in *O*. *tauri* are preferentially encoded by the SOC; these genes are strongly associated with carbohydrate modification and metabolism and their specific regulation was associated with viral resistance.

Other functional categories that were significantly differentially transcribed were related to translation, transcriptional regulation (chromatin remodelling, RNA modification, transcription factors), protein modification and turnover, amino acid transport and modification, and other transporters (Fig 3C). Amino acid biosynthesis genes involved in four different pathways were down-regulated in resistant lines, implicating a decrease in a broad range of amino acids and their downstream metabolites during viral immunity (S4 Table). Ribosomal subunits were also under-expressed suggesting a slowing of growth-related processes. Genes involved in expression regulation were likely involved in maintenance of the virus-resistant state. In particular, histone modification genes were all over-transcribed in the resistant lines pointing to a key role for chromatin restructuring in resistance. Proteasome-mediated protein degradation (ubiquitination) as well as post-translational modification (glutathione-associated and chaperones) were up-regulated and may be part of the viral immunity response or translational-level regulation. Transporters whose substrates were related to undefined small, generally inorganic molecules had mixed regulation with a few cases of transporters with related functions being regulated in opposing directions. For example, a transmembrane phosphate transporter (ostta06g00210) over-expressed while another calcium-dependent phosphate transporter (ostta17g00940) was under expressed (S4 Table). Altered transcription of transporters may modulate the available substrate pool required by OtV5 curbing viral replication. However, these genes show lower significance levels of differential expression and may be involved in processes that are not directly relevant for resistance.

### The bipartite structure and transcription profile of chromosome 19 suggests a regulatory switch

**Fig 5:**
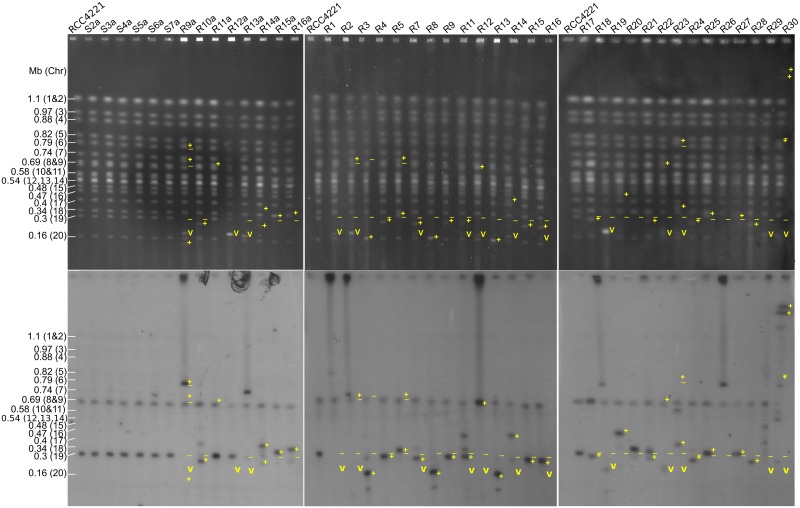
Pulsed-field gel electrophoresis (PFGE) analysis of susceptible and resistant lines ethidium bromide stained (top) and corresponding hybridization (bottom) using a probe for a gene on chromosome 19. Chromosome number and size are indicated on the left. Symbols on the upper gel images show, at positions observed by the ethidium-bromide fluorescence: **−** : absence of a band that was present in controls, **+**: presence of a band that was not present in controls, **v**: presence of a band at the expected size of the OtV5 genome. To aid the comparison, symbols from the upper images of ethidium-stained gels are shown at the same positions on the bottom Southern blot autoradiograph images. Note that since the probe hybridizes to a specific part of chromosome 19, occasionally the band of differing mobility does not correspond to the band identified by radioactive labelling, witnessing fragmentation of chromosome 19 after deletions, insertions, or translocations that may have occurred.

Re-sequencing of the *O*. *tauri* parent line with single molecule PacBio technology was able to resolve a large inverted repeat region (LIRR) on chromosome 19 totalling ~180 kb (Fig 4) that was predicted to exist from previous work [4]. The assembled PacBio contig was 311,428 bp, in agreement with its expected size from gel mobility (Fig 5). Notably, all over-transcribed genes in the resistant lines from the SOC were located in this region (Fig 4). Differentially transcribed genes on other chromosomes (S4 Table) were in some cases adjacent to each other, but were not grouped together as on chromosome 19. Remarkably, transcription of blocks of genes in the LIRR was almost completely suppressed in controls indicating close to half the SOC was silenced in the OtV5-susceptible wild-type state. By contrast, in resistant lines, these genes were highly transcribed, having among the highest mean fragment counts of the differentially transcribed genes (S2 Table). The co-transcription of genes on the LIRR suggests they were under the control of the same regulatory factors.

Re-sequencing was also able to resolve a tandem repeat region (TRR) comprised of ~2,255 bp monomeric repeats sharing 97–99% identity (S1 Fig), which was under-transcribed in resistant lines but still showed some transcription with high sample–sample variance (Fig 4). Although small genes of unknown function (ostta19g00035 and ostta19g00240) occurred in the TRR, the sequences outside of these genes appeared transcribed, suggesting a functional role. The repeats were interrupted at two loci by unique sequences; (1) a 2,232 bp putative terminal repeat retrotransposon in miniature (TRIM) possessing long terminal repeats (LTRs) and (2) a 3,354 bp sequence of unclear origin (S1 Fig). The putative TRIM is likely derived from the complete 5,537 bp LTR retrotransposon, *retrostreo2* on chromosome 8, with which it shares 99% identity in the LTRs. Extensive deletions in the intervening gene sequences suggests the putative TRIM is non-autonomous and is an example of LTR-transposon miniaturisation in *O*. *tauri*, as well as transposition between chromosomes. Tandem repeats interrupted by transposons are hallmarks of centromeric repeats [42,43]. However, the repeat sequence was not found on other chromosomes, as would be expected in centromere recognition sites, leaving the functional significance of the TRR unclear.

We conducted an exploratory analysis of all sequenced transcriptomes (S1 Table) to see if the chromosome 19 genes detected in the differential expression analysis were consistently regulated the same way in independent resistant lines that were not part of the comparative analysis. Hierarchical clustering of the long inverted repeat region (LIRR) gene transcription profiles grouped all susceptible samples separately from the resistant samples (S2A Fig). In particular, two blocks of genes (ostta19g00070–120 and ostta19g000560–610) were transcribed across all independent resistant lines, albeit with variation in transcription levels, but not in the susceptible controls, supporting the LIRR having a shared regulatory mechanism activated by OtV5 infection. By contrast, down-regulated genes were not consistently under-transcribed in all resistant lines (S2B Fig). This indicates over-expression of the LIRR genes was a strong determiner of resistance while under-expression of chromosome 19 genes, including the TRR, was not consistently correlated with resistance.

### Altered carbohydrate synthesis and expression of diverse glycosyltransferases

Several GT genes from chromosome 19 induced in the virus-resistant state were of putative foreign origin (S2 Table) and appeared to be clustered with genes encoding functionalities related to sugar metabolism and modification (Fig 4, left gene map). These genes include a rhamnan synthesis F/Wbx GT (ostta19g00070), family 92 GT (ostta19g00600) and the co-regulated sugar modification enzymes (ostta19g00110, NAD-dependent epimerase/dehydratase; ostta19g00610, CMP-N-acetylneuraminic acid hydrolase).

Ostta19g00070 comprises merged rhamnan synthesis F and Wbx GT domains. The former is allied with bacterial rhamnose-glucose polysaccharide F (RgpF), which in *Streptococcus* transfers rhamnose to the nascent rhamnan backbone, which is incorporated into surface lipopolysaccharide [44]. RgpF domain in *O*. *tauri* is currently the only occurrence in eukaryotes, the species distribution being otherwise restricted to bacteria (Pfam: PF05045). Similarly, Wbx GT domain is found in bacterial gene clusters involved in synthesis of O-antigen, which in *Shigella* comprises repeated monomers of L-rhamnose, D-galacturonic acid and N-acetylgalactosamine residues [45,46]. One putative gene cluster (ostta19g00110–140) comprises a predicted NAD-dependent epimerase/dehydratase, glycoprotein-N-acetylgalactosamine 3-beta-galactosyltransferase, integral membrane galactosyltransferase and triose-phosphate transporter. Strikingly, the 3-beta-galactosyltransferase was associated with sequences from metazoans, while the adjacent genes appear to have homologues in the green lineage. A second putative gene cluster was a CAZy (carbohydrate-active enzymes database) family 92 GT containing a triose phosphate transporter domain (ostta19g00600) adjacent to CMP-N-acetylneuraminic acid hydrolase (ostta19g00610) of metazoan origin. These two genes were putatively involved in the activation of sugars for the synthesis of cell surface sialic acid. In proximity to these clusters of sugar modifcation genes was a predicted FkbM methyltransferase (ostta19g00560), which suggests it has a role in glycan methylation. Two of the up-regulated GTs (ostta19g00130 and ostta19g00630), were predicted to be membrane-associated and could be part of the Golgi mannosyltransferase complex.

Two different GTs and a triose-phosphate transporter on chromosome 19 appear to be recently generated paralogous copies because they share up to 97% amino acid identity (S3 Fig). The paralogues curiously occur in distinct genetic contexts (Fig 4). For example, the previously mentioned 3-beta-galactosyltransferase (ostta19g00120) is adjacent to sugar modification genes and is over-transcribed in resistant lines while the putative paralogue (ostta19g00320) is adjacent to a conserved eukaryotic algae hypothetical protein and was under-transcribed (Fig 4, GT ***a***). In this case, it suggests the same, or similar, base sugar substrate is utilised in both conditions, but alternatively modified in virus resistant lines. How these GTs and triose-phosphate transporters have been duplicated and shifted in this modular fashion is unclear, but the transposon-related genes on chromosome 19 are candidates for mediating these transfers.

The only two GTs that were differentially regulated in resistant lines that were not located on the SOC, were located on chromosome 2. These genes were down-regulated and included glycosyltransferase AER61 (ostta02g00040) and a membrane glycosyltransferase (ostta02g02060). However, other carbohydrate modification and metabolism genes located on chromosome 2 that were up-regulated in resistant lines included a somatomedin B domain-containing protein (ostta02g03940) and carbohydrate glycoside hydrolase (ostta02g04570). The former is involved in binding polysaccharides and the latter in the lysis of O-glycosidic bonds. This implicates a role also for the BOC in carbohydrate modification during viral resistance.

### Transcription of OtV5

As almost all lines were R^P^ at the time of RNA sequencing, we explored the transcriptomes in sequenced samples for evidence of OtV5 transcription (S1 Table). In susceptible controls, a low number of read counts were assigned to few OtV5 genes (S4 Fig); in particular, OtV5_154c and OtV5_159, which correspond to highly transcribed viral specific genes (S3 Table). As susceptible cells had not been exposed to OtV5, apparent transcription of viral genes were taken to be artefacts probably arising from mis-assigned sequencing sample indexes to highly expressed transcripts within the shared sequencing flow cell lane (all samples were multiplexed together). BLAST searches at neither nucleotide levels nor protein levels revealed similarities that would intimate horizontal transfer of these genes between host and virus. As expected, lines that were R^NP^ had no OtV5 transcription and clustering with susceptible controls in their OtV5 transcription profiles (S4 Fig). The R^P^ lines fell into three clusters corresponding to relatively high (five samples), moderate (five samples) and negligible OtV5 transcription (seven samples near-identical to controls) showing that for most R^P^ lines OtV5 transcripts were nearly undetectable.

As some OtV5 genes appeared to be highly transcribed in certain R^P^ lines, we sought to estimate the amount of virus transcription relative to that of the host. The ratio of the mean transcript counts of the most expressed viral gene (OtV5_154c: 5,878) over that of one of the most highly expressed host genes, the ribulose bisphosphate carboxylase small subunit (*rbcS*, ostta18g01880: 49,249), gives 0.12. As a comparison, the transcript abundance ratio of the corresponding genes during *Paramecium bursaria* chlorella Virus 1 (PBCV-1) infection of *Chlorella* was 37, far exceeding host transcription levels [47]. Since relative OtV5 transcription was lower than that of the host, even in R^P^ lines with relatively higher OtV5 transcripts levels, this suggests viral activity was certainly much lower than expected during infection of susceptible *O*. *tauri*.

Nonetheless, the OtV5 genes that were transcribed to a significant level (mean normalised count > 9) covered over 40% of all OtV5 genes. Expressed OtV5 genes were distributed along the entire viral genome with the expression profile varying for each sample (S5 Fig). Although we could not compare the OtV5 expression in R^P^ with control cells in a normal lytic cycle to confirm this, the OtV5 transcription profiles did not indicate specific genes linked to chronic infection, nor a particular stage in viral replication. Crucially, transcripts of capsid proteins were detected (S3 Table) consistent with OtV5 forming virions. This corroborates with results of the virus production assay, which detected infective lytic virions in the medium of R^P^ lines (Fig 2). Apart from virion structure, transcribed OtV5 genes were associated with DNA replication, transcription, amino acid synthesis and carbohydrate modification and metabolism (S3 Table).

Expressed OtV5 carbohydrate metabolism genes included two enzymes involved in the biosynthesis of nucleotide sugars, OtV5_011, a predicted GDP-D-mannose 4,6-dehydratase, and OtV5_042, a putative dTDP-D-glucose 4,6-dehydratase. The homologue of this GDP-D-mannose 4,6-dehydratase in PBCV-1 has a dual functionality acting in both the synthesis of GDP-D-rhamnose and GDP-L-fucose, both of which are monosaccharides present in the capsid glycan but are rare in the host [48–50]. Adjacent to the GDP-D-mannose 4,6-dehydratase is a group 1 GT (OtV5_012c) that was also expressed. The spatial proximity of these genes suggests the glycosyldonor of the group 1 GT is the nucleotide sugar produced by the GDP-D-mannose 4,6-dehydratase. Three additional OtV5 GTs were transcribed including a predicted membrane-localised group 34 GT (OtV5_033), a group 2 diphosphosugar GT (OtV5_035) and a GT with no assigned CAZy group (OtV5_160). All OtV5 encoded GTs had their closest homologues in prasinoviruses indicating they have conserved virus-specific functions.

### Structural rearrangements of chromosome 19 in resistant lines

Given the massive transcriptional changes in chromosome 19 and the known plasticity in outlier chromosome size between *O*. *tauri* strains [17], we examined the karyotypes of all experimental lines by pulsed-field gel electrophoresis (PFGE). Resistant lines showed karyotype changes, most notably as a shift in the size of chromosome 19 (34 of 36 resistant lines tested) while no change was evident in the susceptible controls (Fig 5). The most common changes were an increase of ~20–490 kb (17 lines) or a decrease of ~40–140 kb (13 lines) in the size of chromosome 19. This was likely due in the former case to duplications within chromosome 19 and in the latter, to deletions within, or fission of, chromosome 19. Several resistant lines (R1, R22, R29 and R30) appeared to have lost chromosome 19 or its location was ambiguous. In R9a and R2, there were possible translocations of regions of chromosome 19 to chromosomes 6 and 8 (respectively) or an increase in the size of chromosome 19 with concurrent insertions-deletions in other chromosomes. Indeed, variations in the size of chromosomes other than chromosome 19 were noted in R11a, R3, R5, R22, R23 and R30. More complex changes were also evident, most notably in R30 where bands apparently larger than 1.1 Mb are present that hybridize with the chromosome 19 probe. Interestingly, seven resistant lines (R9a, R13a, R1, R2, R12 R18 and R26) showed densely hybridizing material in the wells, almost always coinciding with a large increase in the size of chromosome 19 (>430 kb). This could correspond to large circular forms that cannot migrate in the PFGE [51], suggesting the extensive changes to chromosome 19 could occur via circular intermediates. However, the presence of circular DNA in the nucleus such as episomes has neither been previously detected nor specifically investigated in *O*. *tauri*.

### Chronic virus production from lysis of a minority of susceptible cells

**Fig 6:**
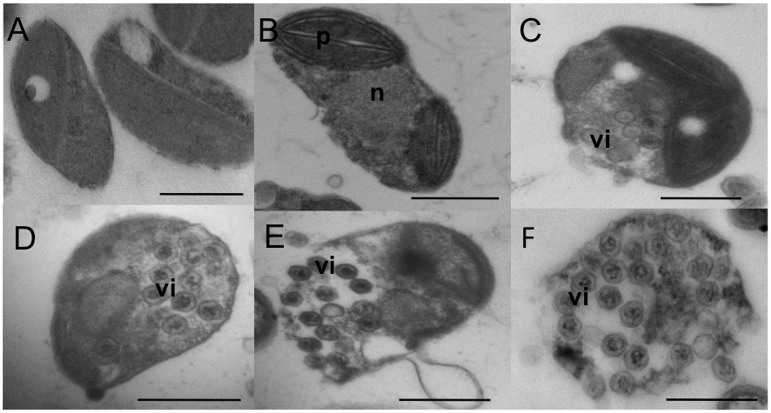
Virus production in two *O*. *tauri* resistant strains. Very few cells (less than 0.5%) show visible viral particles in their cytoplasm. Morphology of lysing R^P^ cells is similar to that of susceptible cells. **(A)** Most of the cells of *O*. *tauri* R^P^2 strain without visible viral particles. (**B)** Dividing *O*. *tauri* R^P^2 strain cell. **(C)** Dividing *O*. *tauri* R^P^3 strain cell with visible intracellular viral particles (vi). **(D)** *O*. *tauri* R^P^3 strain cell with visible intracellular viral particles (vi). **E**. Lysis of an *O*. *tauri* R^P^3 strain cell. **(F)** Lysis of an *O*. *tauri* R^P^2 strain with visible viral particles (vi). The scale bar is 500 nm long.

To investigate the chronic production of OtV5, electron microscopy was performed on two R^P^ lines. This showed a minority of cells (<0.5%) was visibly infected and/or in the middle of lysis (Fig 6) demonstrating viral reproduction proceeded by a typical lytic cycle. Nonetheless, a population crash of the resistant cultures was never observed, with the only loss of lines occurred following antibiotic treatment, so the frequency of lysis was low. Since all lines were re-cloned upon acquiring resistance (Fig 1), the minority of susceptible cells present in R^P^ lines were presumably resistant cells that had switched to a susceptible state.

Production of the OtV5 genome was also observed by PFGE as a ~200 kb band (Fig 5) in 15 of the 19 lines that were R^P^ at that time point. In the four R^P^ lines where the band was not apparent, viral genomes were likely present below the detection limit of DNA staining. The intensities of the viral bands were both greater and less than that of the host chromosomes suggesting, (1) a large variability in amount of viral production between R^P^ lines and (2), given that the number of genomes in the sample is proportional to band intensity, the number of copies of the viral genome was not directly proportional to that of the host. The fact that the OtV5 genome copy number could be much lower than that of the host implies not every cell in the host population was carrying the virus genome. Higher OtV5 band intensities could correspond to viral genomes in virions as well as multiple viral genomes generated during replication in the cell.

### Similar gene functions are conserved in the SOC of all Mamiellales

Outlier chromosomes are found in all Mamiellales sequenced to date, and all SOCs encode genes with a similar set of predicted functions [7], although these genes have very little detectable sequence homology compared to the non-outlier chromosomes. Only 11% of genes on the SOCs of *O*. *lucimarinus* and *O*. *tauri* are orthologues, whereas the average proportion of orthologous genes is 86% on standard chromosomes [52]. We re-analysed the functional genes in all available Mamiellales genomes observing a consistent over-representation of genes involved in carbohydrate transport, synthesis and modification, particularly GTs (Table 1). Prasinoviruses are known to infect all clades of *Ostreococcus*, *Micromonas*, and *Bathycoccus* so far tested [29]. Furthermore, prasinovirus resistance has also been generated in *Bathycoccus prasinos* against BpV2 (*Bathycoccus prasinos* Virus 2) and *Micromonas pusilla* against MpV1 (*Micromonas pusilla* Virus1) in culture [28]. Given that differentially expressed genes on the SOC in *O*. *tauri* was strongly linked to a switch to a virus-immune state, we hypothesize the SOC has a similar role in virus resistance in these other genera of the Mamiellales.

**Table 1:** General characteristics of small outlier chromosomes (SOC) in Mamiellales.

	*O*. *lucimarinus*	*O*. *tauri*	*O*. RCC809	*B*. *prasinos*	*M*. *pusilla* CCMP1545	*M*. RCC299
Chromosome no.	18	19	18	19	19	17
SOC size (kb)†	150	290	102	146	242	195
ORFs	97	101	57	70	125	96
SOC gene density (gene/kb)	0.65	0.35	0.56	0.48	0.52	0.49
Global genome density (gene/kb)	0.58	0.62	0.56	0.52	0.48	0.49
SOC unknown*	49 (51%)	52 (48%)	16 (28%)	39 (56%)	71 (57%)	50 (52%)
Methyltransferase**	11	8	0	7	8	9
Glycosyltransferase	8	14	7	13	5	9
NAD epimerase/dehydratase	6	2	4	2	1	1
Sugar transporter	4	6	2	0	3	3
Other sugar metabolism***	5	10	4	4	0	3
Pyridoxal/vitB6	1	3	0	0	0	0

^†^Size estimate from pulsed-field electrophoresis.

*Including hypothetical (genes having similarity with other Mamiellales SOC genes) and ORFan genes.

**Including protein and/or DNA methyltransferases.

***Including lipopolysaccharide.

## Discussion

### Spontaneous mutation is not enough to explain rapid acquisition of viral resistance

Initially, we designed the experimental strategy to test whether resistant cells may arise spontaneously by mutation, and we planned to look for single nucleotide changes linked to resistance. However, the first visible signs of re-growth of resistant cells appeared about a week after inoculation in all of the cultures. Since cultures do not become visibly green until they reach at least 10^6^ cells.ml^-1^, and assuming the cells have an optimum division rate under these conditions (1.4 divisions.day^-1^ in this growth chamber, M. Krasovec, personal communication), we find that about 1 in 1,000 cells may have become resistant at the time of or just after inoculation with the virus. This, however, may be an underestimation, since after a shock (such as after sub-culturing), there is usually a lag before re-growth. This frequency far exceeds the expected spontaneous mutation rate in *O*. *tauri* since comparison of the re-sequenced *O*. *tauri* genome between 2001 and 2009 revealed the fixation of eight single nucleotide substitutions and two deletions during the approximately 6,000 generations in the lab [4]. We thus preferred an alternative hypothesis, that resistance is induced by the biotic challenge of virus infection, for example by epigenetic modifications affecting gene expression patterns. Since the original culture was clonal, it seemed unlikely that a proportion of host cells was resistant at the moment of inoculation; although we cannot rule out that a reversible regulatory switch to a resistant state occurred in a small proportion of cells analogous to the minority of cells in R^P^ lines that was lysed by OtV5. However, after a period of selection, resistance in the majority of cells appears to be stable since none of the resistant lines were lysed after re-infection over the course of the experiment.

### Extensive alteration of glycosylation state as the principal driver of virus immunity

In several loci, up-regulated genes on the LIRR related to carbohydrate metabolism and modification appeared to be spatially grouped. We speculate genes that are clustered together act on the same carbohydrate synthesis and glycosylation pathway. Notably, genes uniquely expressed in virus-resistant lines might dramatically alter the saccharide composition and glycosylation state of the cell. Several of the highly expressed GTs are associated with synthesis of surface glycans. Surface glycans are known to be important for host–virus interactions, where they mediate initial binding and recognition events of both host cells and pathogens at the cell surfaces [53]. In particular, rhamnan is a polymer important for bacterial host–virus interactions [54,55], and sulphated derivatives of rhamnans are known to have anti-viral activity in mammals [56]. The metazoan-derived CMP N-acetylneuraminic acid hydroxylase (ostta19g00610), in humans makes the influenza A virus receptor, N-acetylneuraminic acid, whose decorations affect receptor specificity [57]. Likewise, glycoprotein-N-acetylgalactosamine 3-beta-galactosyltransferase (ostta19g00120), known to function in the synthesis of extended mucin type O-linked glycans, has been associated with certain immune mediated diseases in humans [58]. Thus, expression of these genes may affect resistance through masking, or altering the *O*. *tauri* surface receptor used by OtV5 to inhibit viral adsorption. However, Thomas et al., [28] observed no statistical difference in OtV5 adsorption to R^P^, R^NP^ and susceptible cells suggesting defective adsorption was not the mechanism for viral resistance. This may be due in part the very low proportion of *O*. *tauri* cells ‘competent’ to OtV5 adsorption at a given time point; even after inoculating susceptible *O*. *tauri* with high titres of OtV5, at most 20% of cells had visibly adsorbed virions despite the fact the majority of cells subsequently lyse [24]. One possible explanation is that the OtV5 receptor is only available during a defined point in host cell cycle, and as *O*. *tauri* cells are not perfectly synchronised in culture [59], adsorption is staggered over time making differences difficult to detect by a standard adsorption assay.

Alternatively, the over-expression of these specific host GTs may curtail OtV5 replication at another stage of the replication cycle, one possible candidate being virion assembly. In the model Chlorovirus, PBCV-1, the major capsid protein is glycosylated at least six sites and the glycans include rare monosaccharides whose biosynthesis genes are virus-encoded (see Piacente et al., [60] and references therein). As OtV5 similarly encodes numerous GTs and homologues to the PBCV-1 sugar metabolism enzymes, many of which are conserved in prasinoviruses (S3 Table), glycoconjugates are likely to be similarly essential to virion structure. Viral resistance may arise by the host switching carbohydrate metabolic pathways, thereby perturbing proper virion formation. This could be mediated, for instance, by feedback inhibition of viral sugar biosynthesis pathways by host generated products, directly altering the viral glycosyldonors/acceptors or simply altering the substrate pool. For example, virally produced GDP-D-rhamnose may be depleted by the host by redirecting it into extracellular rhamnan. Much remains unknown about the glycobiology of both prasinoviruses and Mamiellales and studies of their sugar composition, glycan structure and functional characterisation of GTs will help to pinpoint how the host–virus interaction is suppressed during resistance.

The link between resistance to prasinoviruses and alterations of glycosylation might extend to other Mamiellales, since there are numerous sugar modification genes clustered on the SOC in other members of the Mamiellales with sequenced genomes. They are extremely diverse, with different GT genes in each species, in keeping with the notion that they are important in viral specificity.

### Occasional viral lysis *via* low-level instability in the resistance phenotype?

Thomas et al. [28] reported that chronically infected cells appeared to release viruses by budding, but after examination of many electron micrographs, we found that viral-induced lysis of host cells was occurring, which was further supported by the overall low level of OtV5 transcription in R^P^ lines. R^P^ cell lines are more likely to be unstably resistant, giving rise to a small proportion of revertant susceptible cells. Thus, apparent chronic virus production occurs by lysis of a minority of susceptible cells emerging in a majority of uninfected population at a frequency sufficient to propagate OtV5 over subsequent generations, although the serial cell transfers during the experiment tended towards eventual OtV5 extinction. This further suggests the two apparent kinds of resistance, R^P^ and R^NP^, may reflect observing virus production at the level of a batch culture population and does not correspond to distinct mechanisms of immunity. Over time, revertant susceptible cells arising in cultures where OtV5 were absent could competitively exclude resistant strains, although this was not observed within the almost two-year duration of the study. In keeping with this observation R^P^ cells have been shown to have growth rates similar to wild-type in culture, and differences in fitness could only be shown by competition between co-cultured strains [28].

### Genetic plasticity and transcriptional regulation of the SOC

It is tempting to speculate that the biotic stress induced, by an unknown mechanism, the activation of a transposon that mediated rearrangements in chromosome 19. Activation of LTR-retrotransposons during pathogen attack was observed in tobacco [61] and has since been observed following diverse kinds of biotic or abiotic stresses in diverse organisms (reviewed in [62–64]). This process may lie at the heart of genomic plasticity in response to environmental changes, since genome evolution might be facilitated by bursts of transposon activity [65]. In eukaryotic marine plankton, retrotransposons are probably the most abundantly expressed sequences [66]. The LTR of retrotransposons carry promoters that can affect the transcription of adjacent genes, although the effects of environmental conditions on LTR retrotransposons, which require transcriptional activity for their activation, are complex [62,67,68]. The putative TRIM on chromosome 19 (S1 Fig) may be activated in *trans* by the predicted complete version of this element, *retrostreo2*, on chromosome 8. However, since its reverse transcriptase (ostta08g00390) was down-regulated in resistant lines (S4 Table), it is not a likely candidate for causing changes in the SOC sequence. In addition, the tandem repeats surrounding the retroelement do not look like LTRs, they may be too long (2.3 kb, S1 Fig) and it seems unlikely that transcription of the TRR could influence the LIRR and the other distant and extensive set of resistance genes.

In Mamiellales, DNMT5 was proposed to function as the CpG maintenance methyltransferase [69] as DNMT1 and DNMT3 are lacking, the latter two enzymes being responsible for both *de novo* and maintenance CpG methylation in other organisms [70]. However, extensive CpG methylation occurs in nucleosome linkers and thus apparently functions to guide nucleosome positioning within the extremely compact nucleus. This raises the question of whether or not CpG methylation still plays a role in gene silencing in picoeukaryotes, albeit mediated by an alternative DNMT. A possible MT involved in epigenetic silencing is ostta19g00045, a predicted 5-cytosine methyltransferase located on chromosome 19 that was over-transcribed in the resistant lines (S2 Table). However, if indeed silencing of the LIRR of chromosome 19 in the susceptible control cells occurs by CpG methylation as in plants and animals [70], we would expect decreased activity of *de novo* CpG MT and increased activity of demethylases, which was not the case in this study. This suggests that silencing of chromosome 19 occurs *via* an alternative mechanism.

A more likely candidate is an up-regulated DDE superfamily endonuclease (Fig 4) shared between both outlier chromosomes (ostta19g00165 and ostta02g00275) that may mediate rearrangements. Type II transposons can carry silencer domains in the C-terminal region of their transposases [71] that not only repress their own expression, but can repress expression from adjacent genes, probably by histone modifications that change chromatin conformation. Expression of these transposons can be induced by certain transcription factors. Given that this gene lies at the heart of the over-transcribed region, it might explain both the transcriptional switch and the genome instabilities that we observe. However, it is also possible that an unknown mechanism up-regulating the inverted repeat region leads to de-repression of the predicted endonuclease, leading to the genomic instability observed. In *O*. *lucimarinus* both of the predicted orthologues of ostta19g00165 reside on the chromosome 2, but we have not yet examined the karyotypes of this species after viral infection. Three predicted transcription factors are up-regulated in resistant lines, ostta15g02170, containing a Myb-SANT domain of the REB1 family [72]; ostta01g00380, whose N-terminal regions shows weak similarity to RNA polymerase III subunit delta (BLAST to pfam12657, 1.39e-06) that is important in synthesis of some essential non-coding RNAs [73]; and ostta08g00865. Further experimental investigation would be required to determine whether they play a role in transcriptional activation of the SOC, since these conserved domains are important in numerous kinds of regulatory processes. We might thus speculate that one of these up-regulated transcription factors bind to the ostta19g00165 C-terminal domain after viral attack, inducing the adjacent glycosyltransferases, perhaps by changing chromatin configuration, and activating this transposon. Since the region is a large inverted repeat, an endonuclease target site might also be duplicated and physically distant, perhaps explaining the large size variations observed in this chromosome between independent resistant lines.

Given the presence of numerous repeated regions, and the reported occurrence of homologous recombination in *O*. *tauri* [12], a further alternative might be that the selective pressure for resistance to viruses promotes the growth of relatively rare cells in which genomic rearrangements have arisen by recombination, leading to changed genomic contexts and activation of the inverted repeat region. We have, however, never observed a change in the size of the *O*. *tauri* SOC in culture in our routine laboratory strain RCC4221 over a period of about 20 years at ~300 kb ([5,17] and this work). We were surprised to discover that extensive karyotypic changes had occurred in practically all of resistant lines entailing both increase and decrease in size. This suggests insertions/deletions of genetic material to the SOC after the acquisition of resistance to OtV5 is a consequence of activating resistance genes and not necessarily causative. Future work examining the SOC sequence in virus resistant *O*. *tauri* strains as well as in strains isolated from the environment and monitoring SOC changes during the critical period when resistance is established would inform which genetic changes occur and their relationship to viral resistance.

### Parallels to other host–pathogen systems and evolution of a viral immunity chromosome

Several parallels with other genetic systems that involve biotic interactions between partners of symbionts or pathogens have come to light in our system. In the marine cyanobacterium *Prochlorococcus*, genes involved in resistance to viruses are clustered in hypervariable genomic islands [74] that facilitate rapid evolution of resistance and persistence of the host in some species [75]. As in *Ostreococcus*, in these regions most of these genes may be involved in cell surface interactions. Supernumerary chromosomes, which can vary in size and number between members of the same biological species, have been found in diverse fungal pathogens (reviewed in [76]). They often differ in base composition and structure with other autosomes of the same species, and they can carry genes encoding pathogenicity effectors, probably facilitating the rapid evolution of virulence. In *Nectria haematococca*, for example, pathogenicity effectors are clustered on a dispensable chromosome with an altered GC content that also carries transposons [77] and in *Fusarium* such chromosomes can be transferred horizontally between strains, broadening their host specificity [78]. In *Ostreococcus*, the tables are turned, since it is the host cell that shows genomic plasticity, whereas prasinovirus genomes show less nucleotidic variation between strains than those of their host genomes [26].

We speculate that the evolution of the small outlier chromosome as a viral immunity chromosome in the Mamiellales may have arisen following selection for small genome size, since genome compaction probably led to loss of genes involved in the silencing of viral attack by small RNAs, subsequently forcing the evolution of the defence mechanism typical to this group of algae. Small RNAs are central for epigenetic modifications and genome stability in eukaryotes in general [79,80], and in higher plants mechanisms of resistance to viral infection have been widely studied, where small RNAs have a central role in controlling viral infections (see [77] for a review). Although *Ostreococcus* is in the green lineage, most of the analysed genomes of algae in this group lack detectable homologues of the canonical genes *Argonaut* and *Dicer* thought to be important for their fabrication [81], possibly because it lost these genes during evolution of its extremely small genome that may result from “streamlining” in its marine environment [82]. Loss or gain of the biochemical machinery for fabrication of different kinds of small RNA has led to diversification in eukaryotic systems, and in some well-studied fungal systems loss of gene functions might provide clues about alternative pathways for dealing with viral assault [83].

### Conclusions

To our knowledge, this is the first time that a chromosome specialized in defence against viruses has been described. Our results throw some light on the variability in the size of the SOC observed between different wild-type isolates of the *O*. *tauri* population in coastal north-western Mediterranean Sea [17]. These waters are indeed infested with prasinoviruses [84], and prasinoviruses are similarly abundant in the English Channel, where they infect *Micromonas* spp. [85]. While the precise mechanism for resistance remains to be elucidated by subsequent experimental investigations, we show that challenging cultures with a compatible virus reliably leads to extensive chromosome rearrangements, providing an opportunity to study natural genome evolution in controlled laboratory conditions in *O*. *tauri*. The observed rearrangements in the *O*. *tauri* SOC may be produced by an adaptive mechanism that quickly generates genetic variants able to evade viral lysis. Clustering of the genetic features observed in the SOC helps to explain several of its unusual characteristics. In particular, a faster rate of evolution than the other chromosomes, evidenced in the high level of species-specific genes. The conservation of the same functional gene categories, but not of gene homologues, would also be consistent with rapid “arms-race” evolution against the particular prasinoviruses that infect each species and implies the SOC as a whole has a conserved function in viral immunity.

## Materials and Methods

### Culture conditions and experimental design

The strains used in the experiment were *O*. *tauri* RCC4221 (Roscoff Culture Collection [86] and *Ostreococcus tauri* virus 5 (OtV5). Liquid L1 medium (NCMA, Bigelow Laboratory for Ocean Sciences, USA) was used for culturing throughout and prepared using autoclaved seawater from offshore Banyuls Bay (MOLA station: 42°27'11''N, 3°8'42''E) diluted 10% with MilliQ water and filtered prior to use through 0.22 μm filters. All cultures were maintained under a 12:12 hour light/dark regime in 100 μmol photon m^-2^ s^-1^ white light at 20°C. To grow *O*. *tauri* on a solid medium, molten agarose (1.5%) equilibrated to 60°C was added to cultures to give a final agarose concentration: 0.15% and immediately poured into 9 cm diameter petri dishes. Single *O*. *tauri* colonies were picked from the solid medium using a sterile pipette tip and placed into liquid L1. OtV5 plaques were produced by mixing serial dilutions of OtV5 lysate with *O*. *tauri* RCC4221 culture and growing on solid medium [24]. A stock OtV5 lysate used in all subsequent infections was produced by randomly selecting a plaque, inoculating it into an *O*. *tauri* RCC4221 culture, purifying the resulting lysate (centrifugation at 8,000 g for 20 min and filtration through 0.2 μm) and storing at 4°C.

### Viral susceptibility and virus production tests

To test viral susceptibility, stock OtV5 lysate was added to *O*. *tauri* lines growing in 48-well plates with the precursor susceptible *O*. *tauri* RCC4221 serving as a positive control. Lines were considered susceptible if visible lysis occurred within approximately one week. To test if *O*. *tauri* resistant lines were producing infective virus, an aliquot of the culture medium from actively growing cells was sampled, then centrifuged at 8,000 *g* for 20 min to remove *O*. *tauri* cells. The cell-free medium was added to susceptible *O*. *tauri* RCC4221 growing in 48-well plates. *O*. *tauri* lines were considered to be producing virus if visible lysis occurred in the susceptible line within approximately one week.

### Nucleic acid extraction, sequencing and assembly

DNA was extracted from the initial 1 L culture of the *O*. *tauri* from which all subsequent lines were cloned (Fig 1) using a CTAB extraction method [34]. GATC Biotech performed whole genome sequencing using six single molecule real time (SMRT) cells on the PacBio RS II platform and *de novo* assembly with the Hierarchical Genome Assembly Process (HGAP, Pacific Biosciences). The final updated chromosome 19 sequence was obtained by scaffolding the HGAP-generated unitigs in Geneious [87,88]. Before total RNA extraction, *O*. *tauri* culture lines were subjected to antibiotic treatment to reduce the amount of bacteria in the cultures according to [89]. The antibiotic treated culture was used to inoculate a 200 mL culture for RNA extraction. Cultures were grown to exponential phase (11–32 million cells ml^-1^) and cells harvested by centrifugation at 8,000 *g* for 20 min in a swinging bucket rotor during the first 3 hours of the light cycle. Total RNA was extracted using the Direct-zol RNA kit (Zymo Research). Cell densities for each culture line are recorded in S1 Table. Selection for polyadenylated RNA, library preparation and sequencing was performed at GeT (Génome et Transcriptome, GENOTOUL, Toulouse, France). RNA libraries were sequenced on the Illumina Hi-Seq 2000 platform by multiplexing all samples on a single flowcell lane, which generating paired end reads of 101 bp in length. RNA sequence reads were checked for quality using FastQC [90].

### Differential gene transcription analysis

In order to have a balanced experimental design and to control for batch effects, four OtV5-resistant and four control lines that were from matching RNA extraction batches were selected for differential gene expression analysis (S1 Table). Transcriptome read pairs (fragments) were aligned using TopHat2 [91] (alignment parameters: -i 10 -I 4000, -G) to the annotated genome sequence of *O*. *tauri* RCC4221 [4]. The counts of fragments aligning to each gene was determined using the htseq-count function of HTSeq [92] with parameters (-t CDS -s no -m intersection-nonempty). Exploratory and differential gene expression analyses were performed on fragment count tables using the R package DESeq2 [93]. Hierarchical clustering of the distance between transcriptome profiles after correction for batch effects separated resistant and susceptible lines in two groups (S5 Fig). For differential gene expression, RNA batch number was used as a secondary design variable to adjust for batch effects and the primary variable tested was virus resistance *vs* susceptibility using the DESeq function accepting genes as significantly differentially transcribed with adjusted p-value <0.1.

### Pulsed-field gel electrophoresis and in-gel hybridization

PFGE and in-gel hybridization was conducted as previously described [94]. Briefly, cell cultures were grown to mid-exponential phase, harvested by centrifugation at 8000 *g* for 20 min and cells were resuspended in TE buffer (10 mM Tris-HCl, 125mM EDTA, pH 8) at a density of 8.7 × 10^8^ cells ml^-1^. Electrophoresis was performed in 0.8% agarose gels in 0.5× TBE buffer (44.5 mM Tris, 44.5 mM boric acid, 1 mM EDTA at pH 8) using the CHEF-DR III (Bio-Rad) system. For each sample, 2 mm of plug was loaded into the wells. Electrophoresis was run at 6 V cm^-1^ at 14°C with 120° pulse angle for 15 h with a switch time of 60 s and followed 9 h at a switch time of 90 s. After PFGE, DNA was chemically denatured and dehydrated in a vacuum dryer. PCR was used to amplify a 1681 bp region of the *O*. *tauri* gene ostta19g00640 (5052 bp), located on chromosome 19, with the primer pair 19e_Fw 5´-GCGATGCGGTGCTCTACC-3´ and 19e_Rv 5´CGTGGAGTTATCCCCGAACC-3´ (PCR conditions described in [17]. This gene was chosen as a probe for chromosome 19 because it was consistently transcribed between resistant and susceptible lines (S6 Fig), and thus was not deleted and it has a putative orthologue in the SOC of *O*. *lucimarinus* suggesting a conserved function. After gel purification of the PCR product (Wizard-Prep, kit, Machery-Nagel), the amplicon was randomly labelled with [α-^32^P]CTP (Perkin-Elmer) according to manufacturer’s instructions (Prime-a-Gene kit, Promega) for use as a DNA probe. Dried gels were equilibrated in hybrization buffer (6× SSC, 5× Denhardt's solution, 0.1% (w/v) sodium dodecyl sulphate, 10 μg ml^-1^ tRNA), radiolabelled probe was added, hybridized overnight at 65°C and the gel exposed to radiographic film.

### Accession numbers

Sequence data used in this study can be found in the GenBank data libraries. *O*. *tauri* RCC4221 chromosome sequences are under accession numbers CAID01000001.2 to CAID01000020.2 [4] except for the updated chromosome 19 sequence (this study), which is available from http://wwwphp.obs-banyuls.fr/publications/data/2/. Transcriptome data is available from PRJNA344946. The updated genome sequence and annotation of OtV5 [24] is under accession EU304328.2. Gene models and annotations for *O*. *tauri* RCC4221 are also available from the Online Resource for Community Annotation of Eukaryotes (ORCAE) under *Ostreococcus tauri* V2 [95, 96].

### Transmission electron microscopy

Transmission electron microscopy was performed as previously described in [24] with some modifications. Briefly, 200 mL cultures of *O*. *tauri* in exponential phase were fixed in 1% glutaraldehyde for 30 min and fixed cells were centrifuged for 30 min at 2500 *g* and the cell pellet quickly resuspended in molten (37°C) 1% low melting point agarose (agarose type II, Sigma) and allowed to set in a disposable micropipette (SMI, Emerville, CA, USA). The agarose cell plug was fixed in buffer of one volume of sodium cacodylate (0.4M) in two volumes of L1 medium containing 2.5% glutaraldehyde for 2 h, then washed in the same buffer without glutaraldehyde. Post fixation was performed in 1% OsO4 in sodium cacodylate (0.2M) for 1 h. After two 15 min washes in sodium cacodylate (0.2M), the agarose cell plug was cut into small pieces and dehydrated in ethanol and embedded in Epon 812 resin at 60°C for 48 h. Ultra-thin slices (80–90 nm) were placed on a 300 mesh grid and stained with uranyl acetate for 15 min, followed by lead citrate staining for 2 min then visualised with a Hitachi H 7500 transmission electron microscope.

## Supporting Information

S1 Fig Organisation of the ~37 kb tandem repeat region on chromosome 19. **(A)** Light blue blocks represent 2250–2259 bp repeated monomers with the first and last two nucleotides of the repeat shown if present and the internal magenta arrow indicating the position of the predicted gene, ostta19g00035. Two repeats were interrupted, one by 3354 bp (white rectangle) containing the predicted ORF, ostta19g00240 (black arrow) and the other by a 2232 bp putative TRIM, black bars indicate target site duplication, blue arrows indicate LTRs. **(B)** Zoom in of map comparing the chromosome 19 TRIM to *retrostreo2*, an LTR-retrotransposon on chromosome 8 (5910 bp). Blue arrows are LTRs, purple arrow is the putative POL (ostta08g00390), orange arrow is a gene of unknown function containing a predicted zinc finger domain (ostta08g00400), light green bar is the putative polypurine tract and yellow bar is the putative primer binding site. Red blocks joining the two genomic maps indicate blocks of nucleotide identity (99% in the LTRs, 91% in the central region). The putative genes in the TRIM are likely disrupted.
(TIF)

S2 Fig Heat map of differentially regulated genes on chromosome 19 in *O*. *tauri* samples. RNA fragment counts from alignment to the *O*. *tauri*, organelle and OtV5 genomes were transformed (regularised log_2_) and counts of chromosome 19 genes were used to generate the heat map. **(A)** Up-regulated and **(B)** down-regulated genes from differential gene analysis on chromosome 19 are presented in the order they occur on the genome from left to right (see Fig 4). Virus-susceptible lines are S2a–S5a (blue font), all other samples were OtV5-resistant.
(TIFF)

S3 Fig Multiple sequence alignments of amino acid sequences of putative paralogous genes located on chromosome 19. **(A)** Putative beta1,3-galactosyltransferase, **(B)** putative galactosyltransferase, **(C)** putative glycosyltransferase family 25 and **(D)** putative triose phosphate transporter. Gene identifiers are marked on the left in blue. The positions where the alignment starts and ends are marked on either side of the sequence and the full amino acid sequence length is in parentheses.
(TIFF)

S4 Fig Heat map of OtV5 gene transcription profiles in *O*.*tauri* samples. RNA fragment counts from alignment to the *O*. *tauri*, organelle and OtV5 genomes were transformed (regularised log_2_) and counts of OtV5 genes were used to generate the heat map. Virus-susceptible lines are S2a–S5a (blue font), all other samples were OtV5-resistant. R24 and R26 (yellow) were R^NP^ while all other resistant lines were R^P^ at the time of RNA sequencing.
(TIFF)

S5 Fig Heat map of the sample-to-sample Euclidean distances of *O*. *tauri* transcriptomes. RNA fragment counts from alignment to the *O*. *tauri*, organelle and OtV5 genomes were transformed (regularised log_2_) and sample–sample distances calculated **(A)** without fitting and **(B)** after fitting for the effect of RNA processing batch. Virus susceptible lines are S2a–S5a and resistant lines are R5, R6, R16 and R13a. Sample identifiers from the same RNA processing batch have the same colour.
(TIFF)

S6 Fig Alignment of RNA-Seq reads to the region of chromosome 19 used as a hybridisation probe. Magenta arrows indicate the positions of the forward and reverse primer pair used to generate the probe.
(TIFF)

S1 Table *O*. *tauri* transcriptome sequencing data. (DOCX)

S2 Table Differentially transcribed genes in OtV5-resistant *O*. *tauri* on chromosome 19. (DOCX)

S3 Table OtV5 genes transcribed in resistant *O*. *tauri* lines. (DOCX)

S4 Table Differentially transcribed genes in OtV5-resistant *O*. *tauri* from chromosomes other than chromosome 19. (DOCX)
