# Supplementary figures and images for: A Viral Immunity Chromosome in the Marine Picoeukaryote, Ostreococcus tauri
Source: PLoS Pathog. 2016 Oct 27;12(10):e1005965. doi: 10.1371/journal.ppat.1005965 (PMC5082852; doi:10.1371/journal.ppat.1005965)

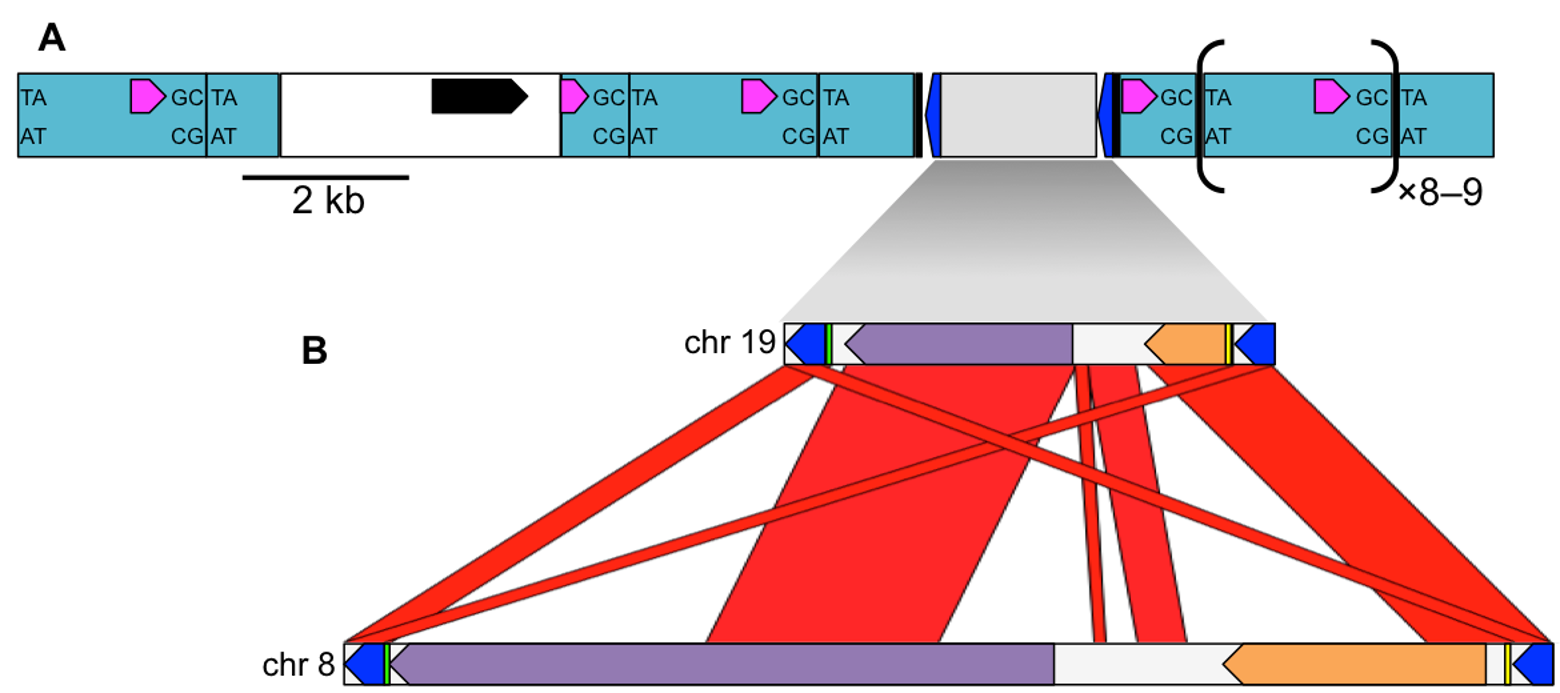

Supplement: S1 Fig — (A) Light blue blocks represent 2250–2259 bp repeated monomers with the first and last two nucleotides of the repeat shown if present and the internal magenta arrow indicating the position of the predicted gene, ostta19g00035. Two repeats were interrupted, one by 3354 bp (white rectangle) containing the predicted ORF, ostta19g00240 (black arrow) and the other by a 2232 bp putative TRIM, black bars indicate target site duplication, blue arrows indicate LTRs. (B) Zoom in of map comparing the chromosome 19 TRIM to retrostreo2, an LTR-retrotransposon on chromosome 8 (5910 bp). Blue arrows are LTRs, purple arrow is the putative POL (ostta08g00390), orange arrow is a gene of unknown function containing a predicted zinc finger domain (ostta08g00400), light green bar is the putative polypurine tract and yellow bar is the putative primer binding site. Red blocks joining the two genomic maps indicate blocks of nucleotide identity (99% in the LTRs, 91% in the central region). The putative genes in the TRIM are likely disrupted. (TIF) [file ppat.1005965.s001.tif]

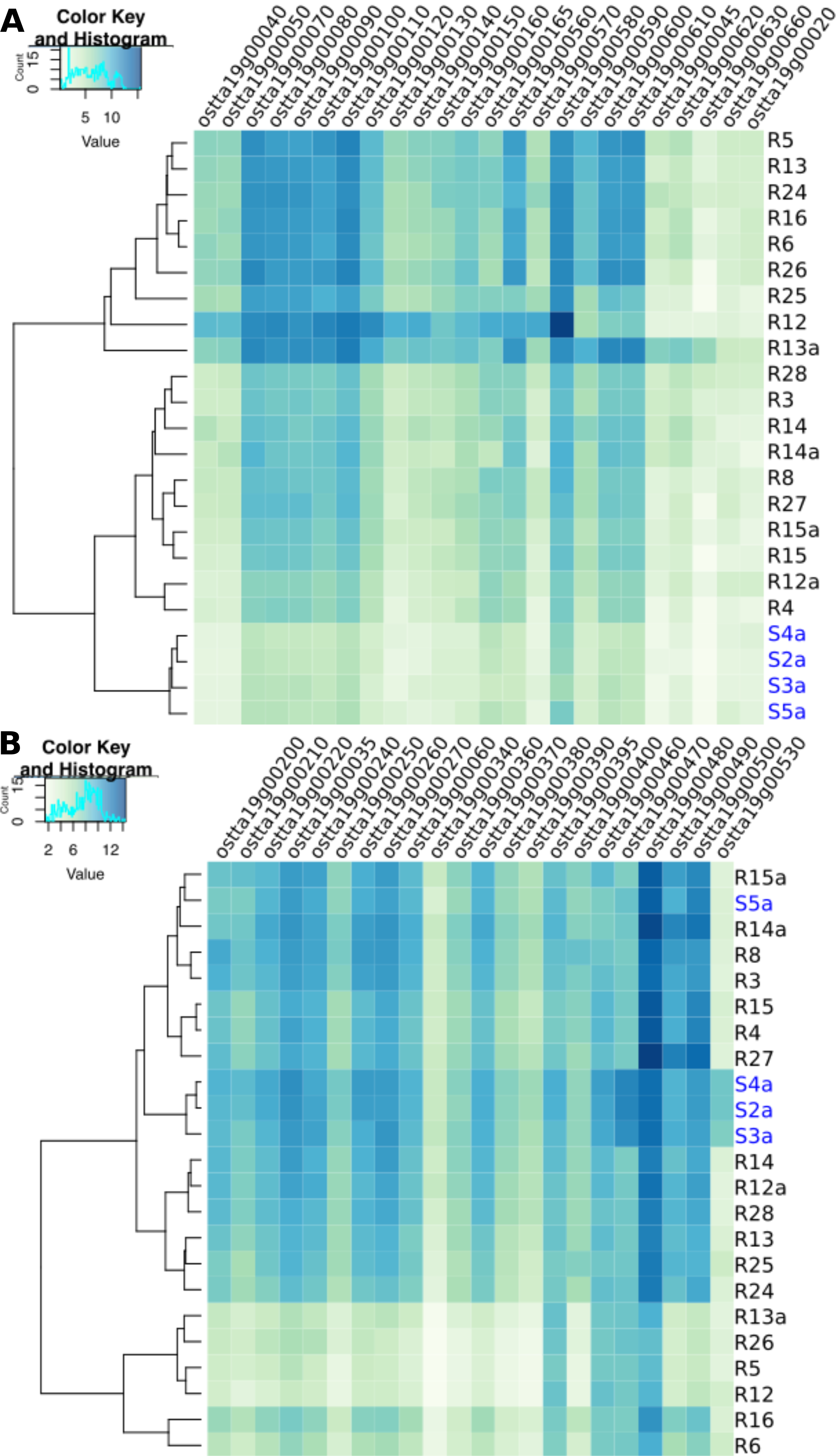

Supplement: S2 Fig — RNA fragment counts from alignment to the O. tauri, organelle and OtV5 genomes were transformed (regularised log2) and counts of chromosome 19 genes were used to generate the heat map. (A) Up-regulated and (B) down-regulated genes from differential gene analysis on chromosome 19 are presented in the order they occur on the genome from left to right (see Fig 4). Virus-susceptible lines are S2a–S5a (blue font), all other samples were OtV5-resistant. (TIFF) [file ppat.1005965.s002.tiff]

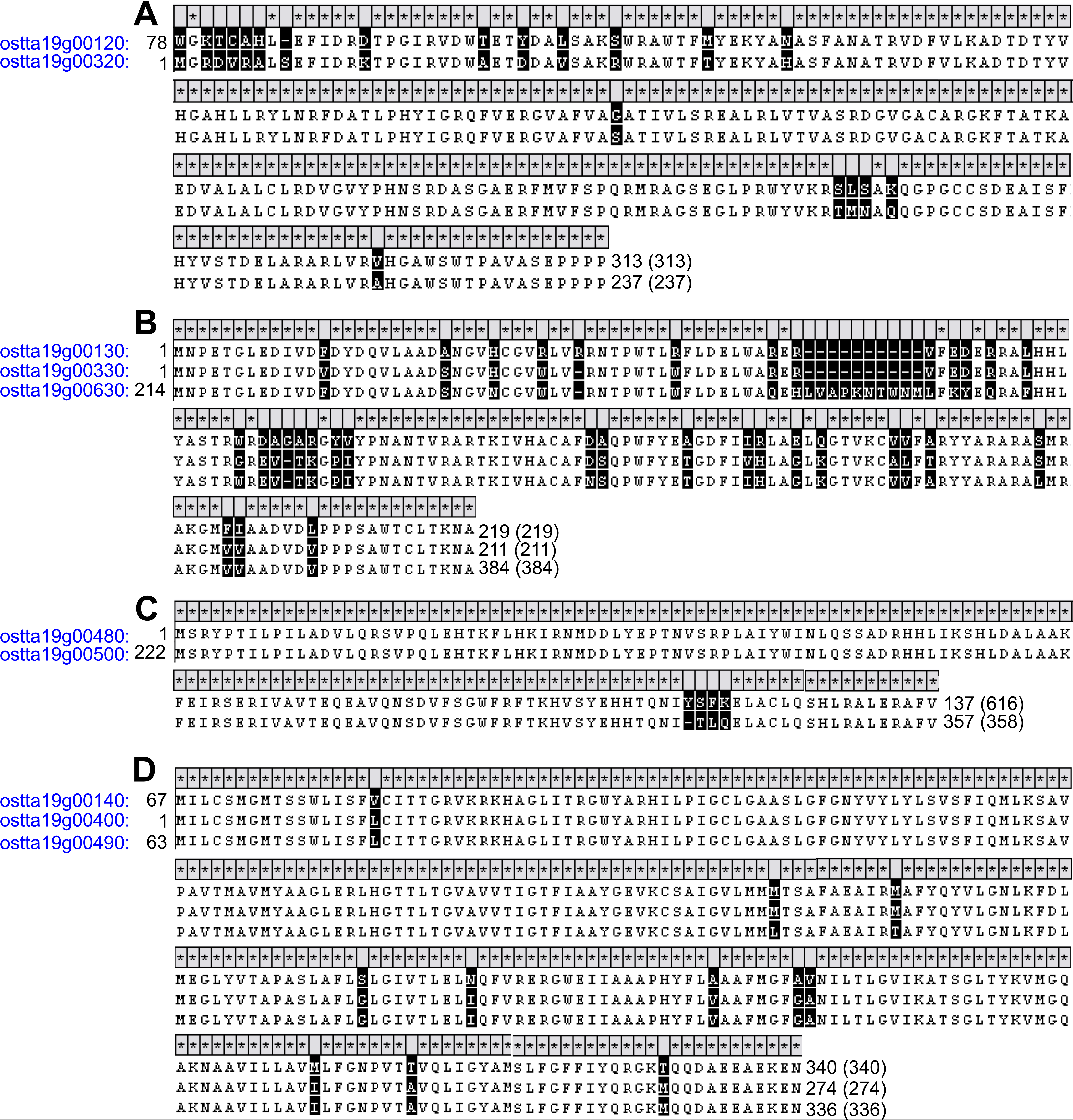

Supplement: S3 Fig — (A) Putative beta1,3-galactosyltransferase, (B) putative galactosyltransferase, (C) putative glycosyltransferase family 25 and (D) putative triose phosphate transporter. Gene identifiers are marked on the left in blue. The positions where the alignment starts and ends are marked on either side of the sequence and the full amino acid sequence length is in parentheses. (TIFF) [file ppat.1005965.s003.tiff]

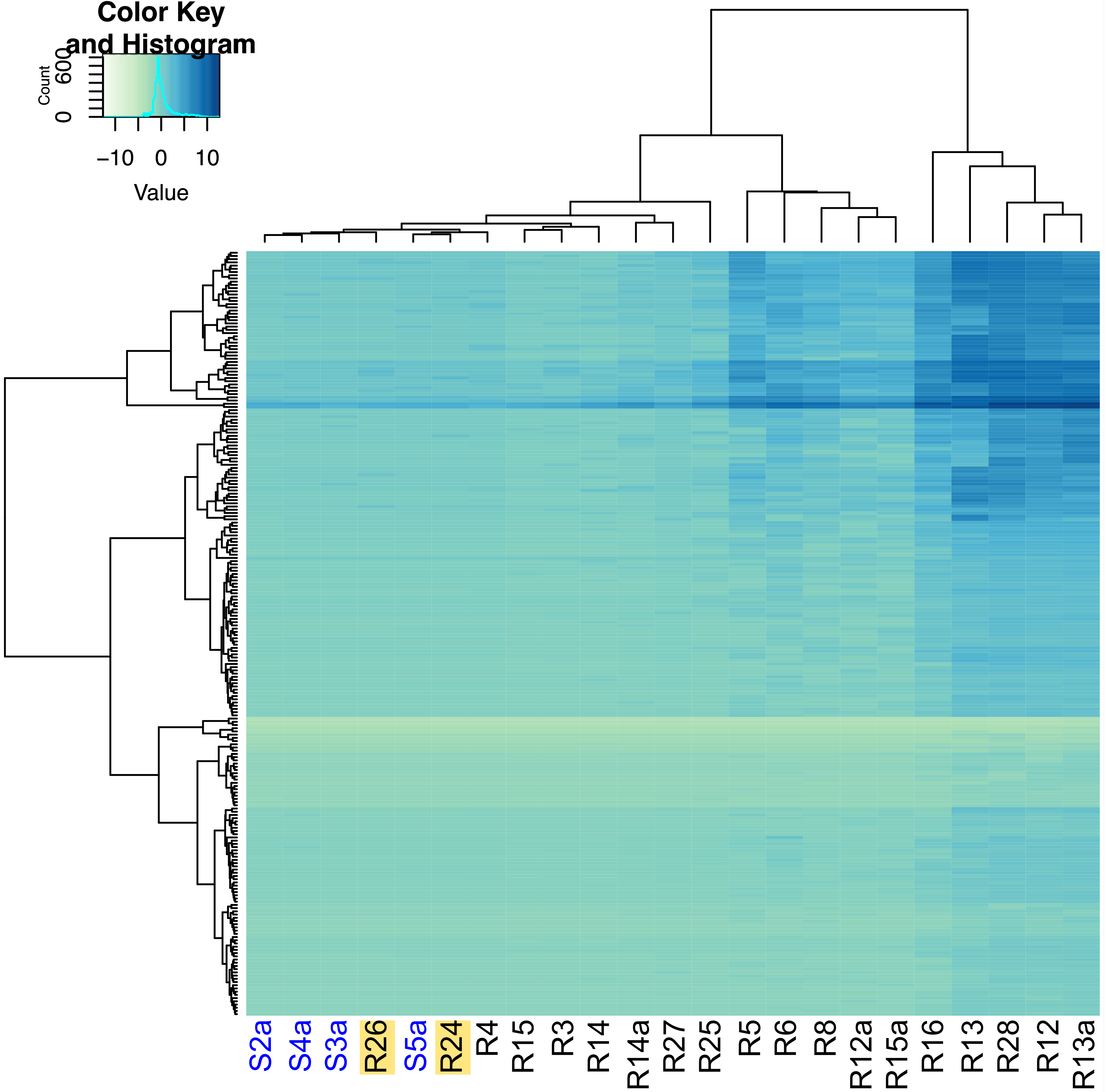

Supplement: S4 Fig — RNA fragment counts from alignment to the O. tauri, organelle and OtV5 genomes were transformed (regularised log2) and counts of OtV5 genes were used to generate the heat map. Virus-susceptible lines are S2a–S5a (blue font), all other samples were OtV5-resistant. R24 and R26 (yellow) were RNP while all other resistant lines were RP at the time of RNA sequencing. (TIFF) [file ppat.1005965.s004.tiff]

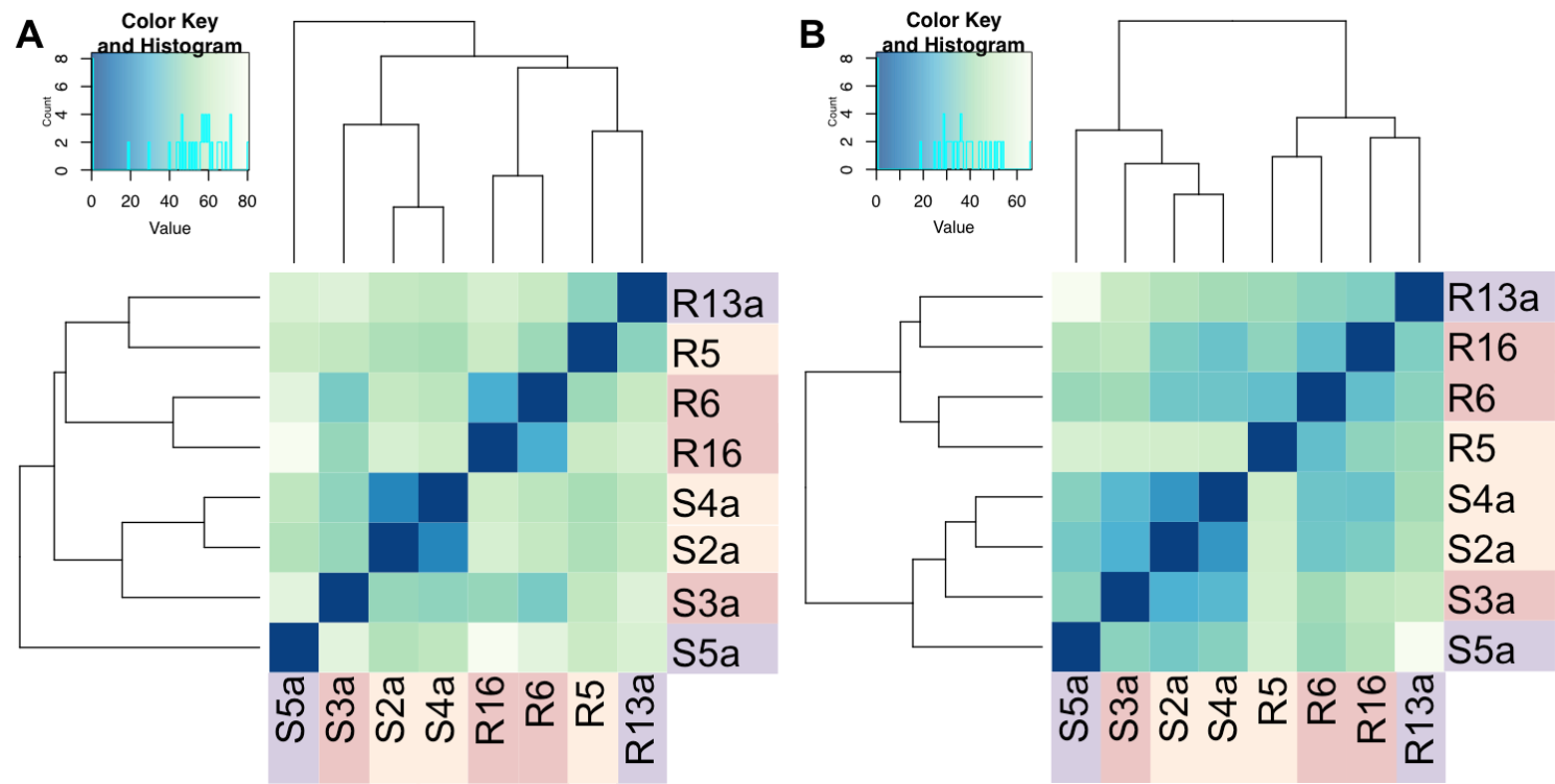

Supplement: S5 Fig — RNA fragment counts from alignment to the O. tauri, organelle and OtV5 genomes were transformed (regularised log2) and sample–sample distances calculated (A) without fitting and (B) after fitting for the effect of RNA processing batch. Virus susceptible lines are S2a–S5a and resistant lines are R5, R6, R16 and R13a. Sample identifiers from the same RNA processing batch have the same colour. (TIFF) [file ppat.1005965.s005.tiff]

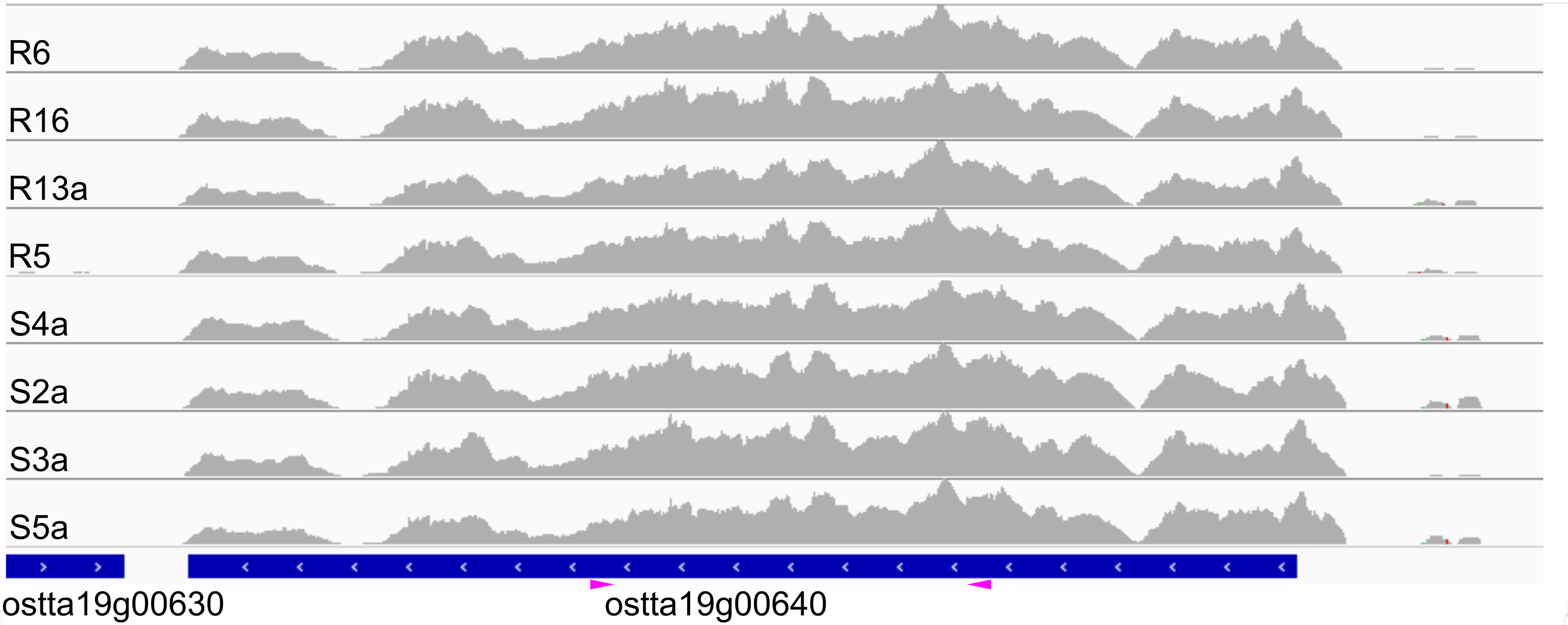

Supplement: S6 Fig — Magenta arrows indicate the positions of the forward and reverse primer pair used to generate the probe. (TIFF) [file ppat.1005965.s006.tiff]
