# Supplementary material for: A Viral Immunity Chromosome in the Marine Picoeukaryote, Ostreococcus tauri
Source: PLoS Pathog. 2016 Oct 27;12(10):e1005965. doi: 10.1371/journal.ppat.1005965 (PMC5082852; doi:10.1371/journal.ppat.1005965)
Supplement: S1 Table — (DOCX) [file ppat.1005965.s007.docx]

**S1 Table.** ***O. tauri* transcriptome sequencing data.** Samples that were used in differential gene transcription analysis are shown in bold.

| ID | OtV5 production† | virus:host  read ratio | RNA batch | *O.tauri* density  (×10^6^ ml^-1^)* | bacterial density  (×10^6^ ml^-1^)* | RNA-Seq read pairs | RNA-Seq aln (%)** |
| --- | --- | --- | --- | --- | --- | --- | --- |
| R3 | + | < 0.0001 | 1 | 25 | 3.4 | 8988620 | 81 |
| R4 | + | < 0.0001 | 1 | 22 | 3.2 | 5006049 | 80 |
| **R5** | **+** | **0.0005** | **3** | **14** | **6.7** | **6566606** | **81** |
| **R6** | **+** | **0.0005** | **4** | **21** | **6.3** | **9137932** | **81** |
| R8 | + | 0.0004 | 2 | 13 | 2.2 | 8140455 | 82 |
| R12 | + | 0.0083 | 2 | 18 | 5.8 | 5876533 | 81 |
| R13 | + | 0.0064 | 1 | 21 | 3.0 | 5861801 | 82 |
| R14 | + | < 0.0001 | 1 | 23 | 4.6 | 11734610 | 84 |
| R15 | + | < 0.0001 | 1 | 22 | 4.2 | 11205833 | 76 |
| **R16** | **+** | **0.0019** | **4** | **21** | **6.9** | **5606235** | **79** |
| R24 | - | < 0.0001 | 2 | 23 | 5.3 | 5159493 | 80 |
| R25 | + | < 0.0001 | 2 | 28 | 4.8 | 7627958 | 81 |
| R26 | - | < 0.0001 | 6 | 27 | 12 | 5674240 | 81 |
| R27 | + | < 0.0001 | 1 | 29 | 6.6 | 4581536 | 84 |
| R28 | + | 0.01 | 2 | 19 | 5.8 | 2815955 | 80 |
| R12a | + | 0.0002 | 1 | 23 | 4.1 | 5605433 | 80 |
| **R13a** | **+** | **0.0098** | **5** | **21** | **44** | **11714339** | **82** |
| R14a | + | < 0.0001 | 6 | 26 | 6.2 | 8613342 | 82 |
| R15a | + | 0.0001 | 1 | 28 | 7.8 | 9022401 | 81 |
| **S2a** | **n/a** | **< 0.0001** | **3** | **27** | **0.99** | **7172079** | **80** |
| **S3a** | **n/a** | **< 0.0001** | **4** | **11** | **0.49** | **5063655** | **82** |
| **S4a** | **n/a** | **< 0.0001** | **3** | **24** | **1.8** | **5613246** | **82** |
| **S5a** | **n/a** | **< 0.0001** | **5** | **32** | **0.23** | **5961561** | **83** |

OtV5-resistant samples identifiers are prefixed with ‘R’, control virus susceptible are prefixed with ‘S’ and sample identifiers containing ‘a’ were produced in a second set. Abbreviations: **+**, positive result; **-**, a negative result; **n/a**, non applicable; virus:host read ratio, number of fragments uniquely aligned to the virus genome divided by fragments aligning to *O. tauri*; RNA batch, samples with the same number were harvested and RNA extracted in parallel (1–6 corresponds to extraction dates 3 Jul., 4 Jul., 6 Jul., 10 Jul., 23 Jul., 12 Aug. 2014). †OtV5 production defined as lysis of *O. tauri* when exposed to cell-free medium from resistant cultures (result from within 25 days around RNA extraction date). *Density of cells just before RNA extraction. **Alignment of properly paired fragments.
