# Supplementary material for: A Viral Immunity Chromosome in the Marine Picoeukaryote, Ostreococcus tauri
Source: PLoS Pathog. 2016 Oct 27;12(10):e1005965. doi: 10.1371/journal.ppat.1005965 (PMC5082852; doi:10.1371/journal.ppat.1005965)
Supplement: S2 Table — (DOCX) [file ppat.1005965.s008.docx]

## S2 Table. Differentially transcribed genes in OtV5-resistant *O. tauri* on chromosome 19. Genes are presented in the order they appear from left to right in Figure 4.

| **Up-regulated** | | | | | | |
| --- | --- | --- | --- | --- | --- | --- |
| **Gene ID** | **Similarity** | **Predicted function** | **Category** | **Mean count** | **log2**  **FC** | **Origin of best matches** |
| ostta19g00040 | pyridoxyl phosphate-dependent transferase, subdomain 2 | aminotransferase: amino acid or amino sugar synthesis | amino acid metabolism | 206 | 4.79 | eukaryotic algae, bacteria |
| ostta19g00050 | pyridoxyl phosphate-dependent transferase, subdomain 1 | aminotransferase: amino acid or amino sugar synthesis | amino acid metabolism | 218 | 7.24 | eukaryotic algae, bacteria |
| ostta19g00070 | rhamnan synthesis F, Wbx glycosyltranferase | rhamnose-glucose polysaccharide synthesis, O-linked glycoprotein synthesis | carbohydrate metabolism | 4691 | 7.56 | bacteria |
| ostta19g00080 | - | unknown | - | 3454 | 6.95 | eukarya |
| ostta19g00090 | - | unknown | - | 3483 | 7.24 | no match |
| ostta19g00100 | - | unknown | - | 3273 | 7.11 | eukarya |
| ostta19g00110 | NAD-dependent epimerase/dehydratase | epimerisation of nucleotide sugars | carbohydrate metabolism | 6998 | 7.57 | eukaryotic algae, plants |
| ostta19g00120 | glycoprotein-N-acetylgalactosamine 3-beta-galactosyltransferase | glycosyltransferase | carbohydrate metabolism | 1029 | 5.86 | metazoans |
| ostta19g00130 | galactosyltransferase | membrane glycosyltransferase | carbohydrate metabolism | 212 | 4.45 | eukaryotic algae, |
| ostta19g00140 | triose-phospate transporter | sugar phosphate transporter | carbohydrate metabolism | 286 | 3.61 | eukaryotic algae |
| ostta19g00150 | - | unknown | - | 269 | 4.16 | no match |
| ostta19g00160 | - | unknown | - | 495 | 5.68 | no match |
| ostta19g00165 | DDE superfamily endonuclease, CENP-B family | DNA binding: function as endonuclease or centromere recognition as part of kinetochore | transposable element-related | 143 | 2.57 | eukarya, putative transposon with putative paralogue on chromosome 2 |
| ostta19g00560 | FkbM methyltransferase | SAM-dependent methylation of proteins, lipids, small molecules, nucleic acids | methyltransferase | 2823 | 7.42 | eukaryotic algae |
| ostta19g00570 | - | unknown | - | 84 | 3.32 | no match |
| ostta19g00580 | - | unknown | - | 4507 | 4.25 | no match |
| ostta19g00590 | - | unknown | - | 1042 | 5.89 | no match |
| ostta19g00600 | triose phosphate transporter, glycosyltransferase family 92 | sugar phosphate transporter, glycosyltransferase | carbohydrate metabolism | 4243 | 6.60 | plants |
| ostta19g00610 | CMP-N-acetylneuraminic acid hydrolase | CMP-N-acetylneuraminate synthesis, putative surface sialic acid modification | carbohydrate metabolism | 4540 | 7.39 | metazoans |
| ostta19g00045 | 5-cytosine methyltransferase | DNA methylation | methyltransferase | 147 | 3.46 | Mamiellales |
| ostta19g00620 | - | unknown | - | 177 | 3.58 | eukaryotic algae |
| ostta19g00630 | galactosyltransferase | membrane glycosyltransferase | carbohydrate metabolism | 39 | 2.12 | eukaryotic algae |
| ostta19g00660 | DUF285, bacterial surface repeat protein | 25–26 tandem peptide repeat containing surface lipoprotein | bacterial surface repeat | 17 | 1.35 | Mamiellales, bacteria |
| ostta19g00020 | epidermal growth factor-like | containing carbohydrate binding, prefoldin chaperone and coagulation factor 5/8 domains | carbohydrate metabolism | 26 | 1.71 | Mamiellales, bacteria |

| **Down-regulated** | | | | | | |
| --- | --- | --- | --- | --- | --- | --- |
| **Gene ID** | **Similarity** | **Predicted function** | **Category** | **Mean count** | **log2**  **FC** | **Origin of best matches** |
| ostta19g00200 | - | unknown | - | 39 | -2.43 | *O. lucimarinus* |
| ostta19g00210 | - | unknown | - | 19 | -2.75 | no match |
| ostta19g00220 | - | unknown | - | 47 | -2.38 | no match |
| ostta19g00230 | alpha1,4-glycosyltransferase domain | putative glycosphingolipid synthesis, Golgi stack | carbohydrate metabolism | 30 | -2.71 | eukaryotic |
| ostta19g00035 | - | unknown, located in tandem repeat | - | 88 | -2.85 | no match |
| ostta19g00240 | - | unknown, located in tandem repeat | - | 47 | -2.99 | no match |
| ostta19g00250 | - | unknown | - | 9 | -2.61 | no match |
| ostta19g00260 | - | unknown | - | 55 | -2.32 | no match |
| ostta19g00270 | FkbM methyltransferase | SAM-dependent methylation of proteins, lipids, small molecules, nucleic acids | methyltransferase | 61 | -1.33 | eukaryotic algae |
| ostta19g00060 | alpha1,4-glycosyltransferase domain | putative glycosphingolipid synthesis, Golgi stack | carbohydrate metabolism | 26 | -1.09 | no match |
| ostta19g00280 | FkbM methyltransferase | SAM-dependent methylation of proteins, lipids, small molecules, nucleic acids | methyltransferase | 24 | -2.83 | eukaryotic algae |
| ostta19g00290 | - | unknown | - | 48 | -2.25 | Mamiellales |
| ostta19g00320 | glycoprotein-N-acetylgalactosamine 3-beta-galactosyltransferase | glycosyltransferase | carbohydrate metabolism | 5 | -2.99 | eukarya |
| ostta19g00340 | - | unknown | - | 1 | -2.8 | paralogues in *O. tauri* chromosome 2 |
| ostta19g00360 | - | unknown | - | 7 | -3.14 | *O. lucimarinus* |
| ostta19g00370 | - | unknown | - | 28 | -1.16 | eukaryotic algae |
| ostta19g00380 | - | unknown | - | 6 | -3.29 | no match |
| ostta19g00390 | - | unknown | - | 4 | -2.79 | no match |
| ostta19g00395 | reverse transcriptase | retrotransposition | transposable element-related | 283 | -1.18 | homologue in *A. thaliana* and Mamiellales |
| ostta19g00400 | triose-phosphate transporter | sugar phosphate transporter | carbohydrate metabolism | 13 | -1.85 | eukaryotic algae |
| ostta19g00460 | C-methyltransferase | SAM-dependent methylation of proteins, lipids, small molecules, nucleic acids | methyltransferase | 252 | -1.29 | Mamiellales, bacteria |
| ostta19g00470 | aminoglycoside phosphotransferase | transfer of nucleosides to phosphosugar | carbohydrate metabolism | 272 | -1.90 | eukarya, bacteria |
| ostta19g00480 | glycosyltransferase family 25 | lipopolysaccharide synthesis, membrane localised | carbohydrate metabolism | 701 | -2.56 | eukaryotic algae, bacteria |
| ostta19g00490 | triose-phosphate transporter | sugar phosphate transporter | carbohydrate metabolism | 68 | -1.24 | eukaryotic algae |
| ostta19g00530 | - | unknown | - | 13 | -2.17 | no match |

Abbreviations: Gene ID, gene identifier; Similarity, significant BLAST hit or INTERPROSCAN protein domain match; Mean count, the mean of the normalised fragment counts of the gene in resistant samples; log2FC, log_2_ fold change in the model comparing transcription in resistants to susceptible controls; Origin of best matches, species of origin of the best BLAST matches.
