# Supplementary material for: A Viral Immunity Chromosome in the Marine Picoeukaryote, Ostreococcus tauri
Source: PLoS Pathog. 2016 Oct 27;12(10):e1005965. doi: 10.1371/journal.ppat.1005965 (PMC5082852; doi:10.1371/journal.ppat.1005965)
Supplement: S3 Table — (DOCX) [file ppat.1005965.s009.docx]

**S3 Table.** **OtV5 genes transcribed in resistant *O. tauri* lines used in differential gene analysis.** These OtV5 genes were expressed at a significant level in R5, R6, R16 and R13a. Genes are ordered as they appear in the OtV5 genome.

| **Gene ID** | **Similarity** | **Predicted function** | **Category** | **Mean**  **counts** | **Origin of best matches** |
| --- | --- | --- | --- | --- | --- |
| OtV5_007c | exonuclease RNase T and DNA polymerase III | 3’–5’ RNA/DNA exonuclease | DNA metabolism | 847 | prasinovirus and bacteria |
| OtV5_008c | - | unknown | - | 52 | no match |
| OtV5_009c | - | unknown | - | 19 | no match |
| OtV5_010c | - | unknown | - | 18 | no match |
| OtV5_011 | GDP-D-mannose 4,6-dehydratase | nucleotide sugar biosynthesis | carbohydrate metabolism | 40 | *Ostreococcus* viruses |
| OtV5_012c | glycosyltransferase group 1 | glycosyltransferase UDP, ADP, GDP or CMP linked sugars to a variety of acceptors | carbohydrate metabolism | 17 | prasinovirus |
| OtV5_015c | - | unknown | - | 51 | no match |
| OtV5_016c | - | unknown | - | 34 | no match |
| OtV5_017 | - | unknown | - | 114 | no match |
| OtV5_018c | - | unknown | - | 627 | no match |
| OtV5_019c | - | unknown | - | 144 | no match |
| OtV5_020c | glycosyltransferase group 1 | glycosyltransferase | carbohydrate metabolism | 347 | prasinovirus and chlorovirus |
| OtV5_021c | - | unknown | - | 30 | no match |
| OtV5_022c | - | unknown | - | 24 | no match |
| OtV5_024c | transcription elongation factor SII | transcription regulation | transcription | 63 | Phycodnaviruses and eukarya |
| OtV5_026 | hypothetical | conserved viral | - | 13 | prasinovirus |
| OtV5_028c | asparagine synthetase B | asparagine synthesis | amino acid metabolism | 20 | prasinovirus and bacteria |
| OtV5_029c | - | unknown | - | 38 | no match |
| OtV5_033c | glycosyltransferase group 34 | membrane glycosyltransferase | carbohydrate metabolism | 10 | Phycodnaviruses and eukarya |
| OtV5_035 | glycosyltransferase group 2 | nucleotide diphosphosugar glycosyltransferase | carbohydrate metabolism | 39 | prasinovirus and bacteria |
| OtV5_036 | thiamine pyrophosphate enzyme | thiamine pyrophosphate binding enzyme | - | 46 | prasinovirus and bacteria |
| OtV5_037c | hypothetical | conserved viral | - | 10 | *Ostreococcus* viruses and bacteria |
| OtV5_041 | DegT/DnrJ/EryC1/StrS aminotransferase | pyridoxal phosphate-dependent aminotransferase: amino sugar or amino acid synthesis | amino acid metabolism | 23 | *Ostreococcus* viruses |
| OtV5_042 | dTDP-D-glucose 4,6-dehydratase | nucleotide sugar biosynthesis | carbohydrate metabolism | 26 | prasinovirus |
| OtV5_050c | hypothetical | conserved viral | - | 10 | Phycodnavirus |
| OtV5_056 | thymidylate synthase, ThyX | FAD-dependent thymine synthesis | DNA metabolism | 19 | prasinovirus, eukarya and bacteria |
| OtV5_058c | ABC-1 domain protein | unknown | - | 23 | prasinoviruses and cyanobacteria |
| OtV5_065 | - | unknown | - | 14 | no match |
| OtV5_072 | major capsid protein | capsid structure | virus structure | 13 | NCLDV |
| OtV5_083 | N-myristoyltransferase | N-terminal protein myristoylation | protein modification | 24 | prasinovirus and eukarya |
| OtV5_084 | mRNA cap guanine-7-methyltransferase | mRNA capping | transcription | 73 | prasinovirus and eukarya |
| OtV5_092 | - | unknown | - | 19 | no match |
| OtV5_098c | similar to ATCV1_Z474R | conserved viral | - | 273 | Phycodnavirus |
| OtV5_099c | similar to NY2A_B560L | conserved viral | - | 124 | Phycodnavirus |
| OtV5_101c | similar to AR158_C590R | conserved viral | - | 21 | Phycodnavirus |
| OtV5_105 | proliferating cell nuclear antigen (PCNA1) | DNA polymerisation co-factor | DNA metabolism | 11 | NCLDV |
| OtV5_119c | viral A-type inclusion protein | unknown | - | 30 | prasinovirus |
| OtV5_124c | - | unknown | - | 160 | no match |
| OtV5_125c | similar to FR483_N327L | conserved viral | - | 132 | Phycodnavirus |
| OtV5_128 | adenine methyltransferase | SWIB/MDM2 cell cycle control | chromatin structure | 151 | NCLDV |
| OtV5_130 | YqaJ-like viral recombinase domain | dsDNA exonuclease | DNA metabolism | 16 | NCLDV |
| OtV5_131c | hypothetical | putative host-derived | - | 21 | *Phycodnaviruses* and eukaryotic algae |
| OtV5_133 | SNF2-like helicase | DNA/chromatin unwinding | transcription | 102 | prasinovirus, chlorovirus and bacteria |
| OtV5_134 | hypothetical | conserved viral | - | 12 | NCLDV |
| OtV5_143 | hypothetical | putative bacteria-derived | - | 16 | *Shewanella baltica* OS155 |
| OtV5_144 | TATA-box binding protein | part of TFIID & TFIIIB | transcription | 162 | NCLDV |
| OtV5_145 | discoidin domain-containing | unknown | - | 38 | *Ostreococcus* viruses |
| OtV5_151c | ser/thr protein kinase | protein phosphorylation | signal transduction | 25 | prasinovirus and plants |
| OtV5_152c | - | unknown | - | 91 | no match |
| OtV5_153c | - | unknown | - | 331 | no match |
| OtV5_154c | 33 kDa *in vitro* translation peptide | most transcribed gene in PBCV-1 | - | 5878 | NCLDV |
| OtV5_155 | - | unknown | - | 24 | no match |
| OtV5_156c | - | unknown | - | 35 | no match |
| OtV5_157c | ribonucleoside-diphosphate reductase | deoxyribonucleotide synthesis | DNA metabolism | 78 | prasinovirus and plants |
| OtV5_158 | - | unknown | - | 102 | no match |
| OtV5_159 | transcription initiation factor IIB | cell cycle control | transcription | 3898 | NCLDV and fungi |
| OtV5_160 | glycosyltransferase | glycosyltransferase | - | 60 | prasinovirus, chlorovirus and bacteria |
| OtV5_161 | similar to FR483_N572L | conserved viral | - | 56 | Phycodnavirus |
| OtV5_162 | mRNA-capping enzyme | RNA processing | transcription | 304 | prasinovirus |
| OtV5_164 | - | unknown | - | 158 | no match |
| OtV5_168c | hypothetical contains bacteriophage/plasmid primase, P4 domain | conserved viral | - | 253 | NCLDV |
| OtV5_169c | - | unknown | - | 125 | no match |
| OtV5_170 | major capsid protein | capsid | virion structure | 22 | NCLDV |
| OtV5_183 | aspartyl/asparaginyl beta-hydroxylase | peptidyl-amino acid modification | amino acid modification | 15 | prasinovirus and bacteria |
| OtV5_184c | haloacid dehalogenase-like hydrolase family protein | unknown | - | 44 | prasinovirus and plants |
| OtV5_185c | DNA ligase | DNA ligation | DNA metabolism | 99 | NCLDV and eukarya |
| OtV5_187c | putative ATP-dependent protease proteolytic subunit | proteolysis | protein metabolism | 15 | Phycodnavirus |
| OtV5_192 | hypothetical protein | conserved viral | - | 12 | *Ostreococcus* viruses |
| OtV5_193 | - | unknown | - | 92 | no match |
| OtV5_201 | - | unknown | - | 37 | no match |
| OtV5_202 | - | unknown | - | 65 | no match |
| OtV5_206c | thymidine kinase | thymidine phospkorylation | DNA metabolism | 9 | prasinovirus and plants |
| OtV5_207 | - | unknown |  | 26 | no match |
| OtV5_210 | dUTPase | reduced UTP incorporation in DNA | DNA metabolism | 29 | prasinoviruses, fungi and metazoa |
| OtV5_211 | - | unknown | - | 37 | no match |
| OtV5_212c | - | unknown | - | 27 | no match |
| OtV5_213c | - | unknown | - | 41 | no match |
| OtV5_214 | fibronection binding protein, DUF814 | putative host derived | - | 12 | prasinovirus and Mamiellales |
| OtV5_219 | DNA polymerase B | DNA replication | DNA metabolism | 43 | NCLDV |
| OtV5_221 | similar to ATCV1_Z045L | conserved viral | - | 25 | Phycodnavirus |
| OtV5_222c | major capsid protein | capsid structure | virion structure | 102 | NCLDV |
| OtV5_223c | DNA topoisomerase II | DNA topoisomerase | DNA metabolism | 177 | prasinovirus and eukarya |
| OtV5_225 | - | unknown | - | 67 | no match |
| OtV5_226 | - | unknown | - | 19 | no match |
| OtV5_227 | - | unknown | - | 102 | no match |
| OtV5_232c | - | unknown | - | 74 | no match |
| OtV5_233 | - | unknown | - | 156 | no match |
| OtV5_234 | - | unknown | - | 44 | no match |
| OtV5_235 | - | unknown | - | 296 | no match |
| OtV5_236 | - | unknown | - | 248 | no match |
| OtV5_237 | - | unknown | - | 34 | no match |
| OtV5_238 | - | unknown | - | 31 | no match |
| OtV5_239 | - | unknown | - | 119 | no match |
| OtV5_240 | hypothetical | unknown | - | 76 | *Ostreococcus* |
| OtV5_241 | - | unknown | - | 65 | no match |
| OtV5_243 | - | unknown | - | 30 | no match |

Abbreviations: Gene ID, gene identifier; Similarity, significant BLAST hit; Mean counts, the mean of the normalised fragment counts mapping to the gene in resistant samples; Origin of best matches, species of origin of the best BLAST matches, NCLDV, Nuclear Cytoplasmic Large DNA Virus.
