# Supplementary material for: A Viral Immunity Chromosome in the Marine Picoeukaryote, Ostreococcus tauri
Source: PLoS Pathog. 2016 Oct 27;12(10):e1005965. doi: 10.1371/journal.ppat.1005965 (PMC5082852; doi:10.1371/journal.ppat.1005965)
Supplement: S4 Table — (DOCX) [file ppat.1005965.s010.docx]

**S4 Table. Differentially transcribed genes in OtV5-resistant *O.tauri* from chromosomes other than chromosome 19.**

| **Gene ID** | **Similarity** | **Predicted function** | **Category** | **Mean counts** | **log2**  **FC** | **Origin of best matches** |
| --- | --- | --- | --- | --- | --- | --- |
| ostta01g00380 | transcription factor IIIC, agenet-like domain | transcription regulation, RNA-binding | transcription factors | 85 | 1.15 | *O. lucimarinus* |
| ostta01g00540 | drug/metabolite transporter | integral membrane transporter | transporter | 243 | -0.9 | Mamiellales and bacteria |
| ostta01g00670 | peroxiredoxin | glutathione-dependent reduction of phospholipid hydroperoxides | protein modification and turnover | 262 | 1.03 | eukarya |
| ostta01g03820 | - | unknown | - | 70 | 1.50 | *O. lucimarinus* |
| ostta01g04230 | aldehyde dehydrogenase | aldehyde dehydrogenase | general function prediction | 49 | 1.13 | eukarya and bacteria |
| ostta01g05220 | - | unknown | - | 36 | 1.29 | Mamiellales and plants |
| ostta01g05960 | C2 calcium-dependent membrane targeting module | signal transduction or membrane trafficking | signal transduction | 1131 | 0.97 | Mamiellales and heterotrophic protists |
| ostta01g06030 | cyclin-like | control of cell division | cyclin-like | 10 | 1.26 | Mamiellales and plants |
| ostta02g00040 | glycosyltransferase AER61 | glycosyltransferase | carbohydrate metabolism | 648 | -0.62 | eukarya |
| ostta02g00085 | - | unknown | - | 111 | 2.19 | no matches |
| ostta02g00210 | aspartyl/asparaginyl beta-hydroxylase | peptidyl-amino acid modification | amino acid metabolism | 488 | -0.59 | eukaryotic algae |
| ostta02g00275 | DDE superfamily endonuclease, CENP-B family | DNA binding: function as endonuclease or centromere recognition as part of kinetochore | transposable element-related | 53 | 1.37 | eukarya, copy on chromosome 19 |
| ostta02g02060 | galactosyltransferase 34 | membrane glycosyltransferase | carbohydrate metabolism | 0 | -1.21 | Mamiellales |
| ostta02g02065 | - | unknown | - | 15 | -2.92 | Eh Virus 86 |
| ostta02g02080 | - | unknown | - | 134 | -2.02 | multiple copies in *O. tauri* SOC and BOC |
| ostta02g02460 | phosphate transporter | transmembrane transporter of inorganic phosphate | transporter | 7291 | -0.78 | Mamiellales, eukaryotic algae and prasinovirus |
| ostta02g02870 | glyceraldehyde 3-phosphate dehydrogenase, NAD(P) binding domain | oxidoreductase activity, acting on the aldehyde or oxo group of donors | carbohydrate metabolism | 254 | -0.91 | eukaryotic algae and cyanobacteria |
| ostta02g02930 | zinc finger, CCCH-type | nucleic acid binding | zinc finger, CCCH-type | 27 | 1.27 | Mamiellales and fungi |
| ostta02g03370 | cyclic nucleotide-dependent serine/threonine protein kinase | protein phosphorylation | signal transduction | 124 | 1.01 | eukaryotic algae and oomycetes |
| ostta02g03460 | pyridoxal phosphate-dependent transferase | aminotransferase: amino acid or amino sugar synthesis | amino acid metabolism | 457 | -0.64 | Mamiellales and metazoans |
| ostta02g03640 | chorismate synthase | terminal step in shikimate pathway, biosynthesis of aromatic amino acids | amino acid metabolism | 1054 | -0.59 | eukaryotic algae and plants |
| ostta02g03940 | somatomedin B domain | involved in polysaccharide binding | carbohydrate metabolism | 70 | 1.24 | Mamiellales |
| ostta02g04420 | - | unknown | - | 351 | -0.78 | *O. lucimarinus* |
| ostta02g04570 | glycoside hydrolase | hydrolysis of O-glycosidic bonds | carbohydrate metabolism | 261 | 0.97 | eukaryotic algae and plants |
| ostta02g04600 | glycine-tRNA ligase, | attachment of an amino acid to its cognate transfer RNA | translation | 953 | -0.60 | eukaryotic algae and plants |
| ostta03g00240 | EF-Hand 1, calcium-binding site, concanavalin A-like lectin/glucanase domain, galactose binding domain | calcium and sugar binding protein | general function prediction | 736 | 1.20 | Mamiellales |
| ostta03g00590 | major facilitator superfamily domain | transport of small solutes | transporter | 182 | -0.62 | eukaryotic algae |
| ostta03g00670 | - | unknown | - | 19 | 1.32 | *O. lucimarinus* |
| ostta03g01210 | thiopurine S-methyltransferase | SAM dependent methylation of aromatic and heterocyclic sulphydryl compounds | methyltransferase | 233 | -1.00 | eukarya |
| ostta03g01310 | - | unknown | - | 23 | 1.36 | *O. lucimarinus* |
| ostta03g01830 | WD40/YVTN repeat-like-containing domain | protein-protein interactions involved in signal transduction and transcription regulation to cell cycle control and apoptosis | general function prediction | 16 | 1.13 | eukarya |
| ostta03g01960 | defective-in-cullin neddylation protein | neddylation of cullin components of ubiquitin ligase complexes, proteolysis | protein modification and turnover | 62 | 0.93 | Mamiellales and plants |
| ostta03g02090 | major royal jelly-related | unknown | - | 148 | 0.96 | Mamiellales |
| ostta03g02500 | mitochondrial substrate/solute carrier | transport across mitochondrial or organelle membrane | transporter | 3800 | 0.82 | eukaryotic algae |
| ostta03g02780 | - | unknown | - | 258 | 1.52 | eukaryotic algae |
| ostta03g02950 | tetratricopeptide repeat-containing domain, protein arginine N-methyltransferase | protein-protein interaction, SAM dependent methylation of histones | chromatin structure | 54 | 1.49 | eukaryotic algae |
| ostta03g03860 | dihydroxy-acid dehydratase  aconitase/3-isopropylmalate dehydratase | dehydratation activity,  interconversion of isocitrate and citrate | amino acid metabolism | 621 | -0.75 | eukaryotic algae |
| ostta04g00290 | dimeric alpha-beta barrel | unknown | - | 59 | -1.43 | Mamiellales and bacteria |
| ostta04g00370 | ankyrin repeat | protein-protein interactions | ankyrin repeat | 1117 | 1.05 | Mamiellales and plants |
| ostta04g01340 | NADH-azoreductase, FMN-dependent | hydrolysis of azo bond in aromatic azo compounds | general function prediction | 372 | -0.74 | bacteria |
| ostta04g02540 | ubiquitin activating enzyme | adenylation of ubiquitin | protein modification and turnover | 41 | 1.36 | Mamiellales and plants |
| ostta04g02860 | - | unknown | - | 1537 | -0.87 | Mamiellales and plants |
| ostta04g03830 | - | unknown | - | 60 | 1.06 | no match |
| ostta05g02426 | prefoldin | molecular chaperone | protein modification and turnover | 2000 | 0.69 | Mamiellales |
| ostta05g03630 | DUF448 | unknown | - | 34 | 1.33 | Mamilellales and bacteria |
| ostta05g03660 | tetratricopeptide-like helical domain, DnaJ domain | protein-protein interactions, molecular chaperone | protein modification and turnover | 71 | 1.19 | eukarya |
| ostta05g03910 | phosphoesterase-like | phosphoesterase activity | general function prediction | 61 | 1.19 | eukaryotic algae |
| ostta06g00210 | major facilitator superfamily domain | transport of small solutes | transporter | 98 | 1.14 | eukaryotic algae |
| ostta06g00220 | uncharacterised protein family UPF0047 | unknown | - | 30 | 1.21 | eukaryotic algae |
| ostta06g00530 | chaperone-like protein of POR1-like | regulation of protochlorophyllide oxidoreductase stability and function in the chloroplast | protein modification | 430 | -0.85 | Mamiellales and plants |
| ostta06g01630 | peptidase M50, putative membrane-associated zinc metallopeptidase | membrane associated peptidase | general function prediction | 279 | 0.98 | eukaryotic algae and plants |
| ostta06g02350 | - | unknown | - | 32 | 1.17 | *O. lucimarinus* |
| ostta06g03490 | - | unknown | - | 72 | 1.19 | no hits |
| ostta06g03630 | SWI/SNF-related actin-dependent regulator of chromatin | chromatin modification | chromatin structure | 64 | 0.95 | eukarya |
| ostta06g03840 | rhodanese domain | unknown | general function prediction | 170 | 1.25 | Mamiellales and plants |
| ostta07g00030 | protein arginine N-methyltransferase | SAM-dependent methylation of proteins | protein modification and turnover | 100 | 0.98 | eukaryotic algae |
| ostta07g00140 | - | unknown | - | 156 | -0.65 | Mamiellales and plants |
| ostta07g00240 | protein of unknown function DUF4539 | unknown | - | 45 | 1.12 | eukarya |
| ostta07g04160 | - | unknown | - | 455 | 1.56 | eukaryotic algae and plants |
| ostta07g04360 | NAD(P) binding domain | unknown | - | 729 | 0.70 | eukaryotic algae and plants |
| ostta08g00390 | ribonuclease H domain, reverse transcriptase | retrotransposition | transposable element-related | 182 | -0.66 | eukaryotic algae, plants and fungi |
| ostta08g00400 | zinc finger, CCCH-type | nucleic acid binding | zinc finger | 303 | 1.88 | homolog in *O. lucimarinus* |
| ostta08g00865 | cold-shock domain containing | binding of single stranded nucleic acid, transcription regulation | transcription | 216 | 0.95 | eukaryotic algae and plants |
| ostta08g01650 | homoserine kinase | homoserine kinase activity involved in threonine metabolism | amino acid metabolism | 252 | -0.84 | eukaryotic algae and plants |
| ostta08g01880 | zinc finger, CCCH-type | nucleic acid binding | zinc finger | 3161 | -1.03 | Mamiellales and plants |
| ostta08g01920 | histone H2B | DNA binding nucleosome component | chromatin structure | 13 | 1.18 | Mamiellales |
| ostta08g01970 | RAP domain | unknown | general function prediction | 1292 | -0.54 | eukaryotic algae and plants |
| ostta08g02530 | glutathione-dependent formaldehyde activating enzyme/centromere protein V | condensation of formaldehyde and glutathione to S-hydroxymethyl  glutathione | protein modification and turnover | 41 | 0.99 | eukaryotic algae and bacteria |
| ostta08g03000 | DUF285, bacterial surface protein 26-residue repeat | 25–26 tandem peptide repeat containing surface lipoprotein | bacterial surface repeat | 998 | 1.35 | eukaryotic algae and bacteria |
| ostta08g03010 | DUF285, bacterial surface protein 26-residue repeat | 25–26 tandem peptide repeat containing surface lipoprotein | bacterial surface repeat | 1505 | 1.21 | eukaryotic algae and bacteria |
| ostta08g03660 | glutathione S-transferase | glutathionylation | protein modification and turnover | 139 | 1.12 | eukaryotic algae |
| ostta08g04160 | ankyrin-repeat containing | protein-protein interactions | ankyrin repeat | 29 | 1.22 | Mamilellales |
| ostta09g00430 | U box domain, WD40/YVTN repeat-like-containing domain | ubiquitination, protein-protein interactions involved in signal transduction and transcription regulation to cell cycle control and apoptosis | chromatinn structure | 120 | 0.91 | eukaryotic algae and bacteria |
| ostta09g00500 | haem peroxidase | peroxidase activity | general function prediction | 41 | 1.20 | plants |
| ostta09g00590 | - | unknown | - | 78 | 1.64 | eukaryotic phytoplankton and metazoans |
| ostta09g01615 | ABC-type transporter | active transport of molecules across a membrane | transporter | 3450 | -1.46 | eukaryotic phytoplankton and plants |
| ostta10g00130 | fasciclin-like domain containing | extracellular domain, putative cell adhesion or signalling activity | general function prediction | 102 | -1.06 | eukarya and bacteria |
| ostta10g01260 | beta lactamase domain containing | hydrolase activity | general function prediction | 840 | -0.92 | eukaryotic algae |
| ostta10g01370 | fumarate hydratase | conversion of fumerate to L-malate | general function prediction | 43 | 1.15 | Mamiellales, and bacteria |
| ostta10g01500 | - | unknown | - | 378 | -0.73 | eukaryotic algae, plants |
| ostta10g01630 | - | unknown | - | 26 | 1.27 | eukarya |
| ostta10g02740 | alpha/beta hydrolase fold containing | oxidoreductase activity | general function prediction | 77 | 0.85 | Mamiellales, plants and bacteria |
| ostta11g01780 | - | unknown | - | 194 | 0.93 | multiple copies in *O. tauri* |
| ostta11g02820 | dihydrolipoamide dehydrogenase, pyridine nucleotide-disulphide oxidoreductase | FAD-dependent oxidoreductase activity | general function prediction | 2862 | 1.41 | Mamiellales and plants |
| ostta11g03180 | - | unknown | - | 441 | -1.41 | *O. lucimarinus* |
| ostta12g00730 | 5.8S rRNA | translation | translation | 3666 | -1.07 | Mamilellales |
| ostta12g00750 | small subunit rRNA | translation | translation | 33119 | -1.08 | Mamilellales |
| ostta12g01070 | cyclophilin-type peptidyl-prolyl cis-trans isomerase | protein peptidyl-prolyl isomerization, acceleration of protein folding | protein modification and turnover | 302 | 0.87 | eukaryotic algae and plants |
| ostta12g01850 | endoplasmic reticulum oxidoreductin,  cytochrome b5-like heme/steroid binding domain | formation of disulfide bonds of proteins, oxidation-reduction of succinate | protein modification and turnover | 282 | 0.77 | eukaryotic algae and fungi |
| ostta12g02830 | translation initiation factor IF2/IF5 | stimulation of initiation of translation | protein modification and turnover | 595 | -0.76 | eukaryotic algae and plants |
| ostta13g01770 | peptidase C48 (SUMO/Sentrin/  Ubl1), MULE-transposase domain | ubiquitin-like protease | protein modification and turnover | 8 | -2.38 | Mamilellales and metazoans |
| ostta13g02380 | RNA recognition motif containing, nucleotide binding alpha-beta plait domain | recognition of ssRNA, may function in RNA stability, alternative splicing, component of ribonucleoprotein | general function prediction | 70 | 1.27 | Mamielleles and plants |
| ostta14g01760 | RWP-RK domain containing protein | plant regulatory protein | general function prediction | 1730 | 1.63 | Mamielleles and plants |
| ostta14g01890 | malate/L-lactate dehydrogenase | oxidation of malate to oxaloacetate | energy production and conversion | 10927 | 0.90 | eukaryotic algae |
| ostta14g02970 | isocitrate/  isopropylmalate dehydrogenase | oxidation/reduction of isocitrate, isopropylmalate or tartrate | carbohydrate metabolism | 878 | -0.69 | Mamielleles, Cyanobacteria and metazoan |
| ostta15g00340 | - | unknown | - | 80 | 0.89 | Mamiellales |
| ostta15g01770 | triose-phosphate transporter domain | sugar phosphate transport | carbohydrate metabolism | 628 | 1.27 | plants |
| ostta15g02030 | Sec20 | secretory pathway associated glycoprotein | secretion | 132 | 1.01 | plants |
| ostta15g02170 | SANT/Myb-like domain | DNA binding | chromatin structure | 4456 | 1.03 | plants |
| ostta15g02920 | P2X purinoreceptor | transmembrane cation transporter responding to purine nucleotides | transporter | 189 | 1.19 | eukaryotic algae |
| ostta16g01530 | dimeric alpha-beta barrel | domain involved in diverse functions | general function prediction | 672 | 1.00 | eukaryotic algae |
| ostta16g01630 | hypothetical | unknown | - | 379 | 0.87 | Mamiellales |
| ostta16g02160 | hypothetical | unknown | - | 528 | -1.33 | *O. lucimarinus* |
| ostta16g02320 | hypothetical | unknown | - | 111 | -0.81 | Mamiellales |
| ostta16g02375 | pseudouridine synthase I, TruA | pseudouridine synthesis | RNA modifcation | 280 | -0.71 | plants |
| ostta16g02560 | argininosuccinate synthase | arginine synthesis | amino acid metabolism | 1168 | -0.79 | eukaryotic algae, bacteria |
| ostta17g00190 | bacterial surface protein 26-residue repeat | 25–26 tandem peptide repeat containing surface lipoprotein | bacterial surface repeat | 6 | -1.31 | Mamiellales, bacteria |
| ostta17g00630 | - | unknown | - | 627 | 0.98 | no match |
| ostta17g00795 | drug/metabolite transporter | membrane transporter | transporter | 400 | -1.05 | eukaryotic algae |
| ostta17g00940 | domain of unknown function DUF221 | Ca-dependent putative phosphate transporter, membrane protein | transporter | 38 | 1.10 | eukaryotic algae |
| ostta17g02150 | mitochondrial substrate/solute carrier | mitochrondrial membrane transporter | transporter | 1416 | 1.56 | Mamiellales, oomycetes |
| ostta18g00210 | post-SET domain | potential histone methyltransferase | chromatin structure | 45 | 1.03 | Mamiellales and insects |
| ostta18g00950 | - | unknown | - | 227 | 1.14 | *O. lucimarinus* |
| ostta18g01010 | Orn/DAP/Arg decarboxylase 2 | pyridoxyl-dependent decarboxylase of either lys arg, ornithine or related substrates, amino acid synthesis | amino acid metabolism | 713 | -0.66 | eukaryotic alga, bacteria |
| ostta18g01100 | PUA-like domain, SAM-dependent methyltransferase | RNA binding, SAM-dependent methylation of RNA | RNA modification | 155 | 1.76 | plants |
| ostta20g00030 | ankyrin repeat, acyl transferase | protein-protein interaction, fatty acid synthesis | fatty acid synthesis | 32 | 1.12 | eukaryotic algae |
| ostta20g00090 | - | unknown | - | 12 | 1.24 | no match |
| ostta20g00110 | peptidase M24, methionine aminopeptidase | contains DNA binding domain, proteolysis, potential transcriptional regulator | protein modification and turnover | 887 | -0.62 | plants |
| ostta20g00170 | phytanoyl-CoA dioxygenase | phytanic acid oxidation | general function prediction | 1850 | 1.21 | Eukaryotic algae |
| ostta20g00180 | serine/threonine protein kinase | protein phosphorylation | signal transduction | 18 | 1.27 | Eukaryotic algae, bacteria |
| ostta20g00730 | translation elongation/initiation factor/ribosomal, beta-barrel | protein translation | translation | 50 | 1.24 | plants |

Abbreviations: Gene ID, gene identifier; Similarity, significant BLAST hit or INTERPROSCAN protein domain match; Mean count, the mean of the normalised fragment counts of the gene in resistant samples; log2FC, log_2_ fold change in the model comparing transcription in resistants to susceptible controls; Origin of best matches, species of origin of the best BLAST matches.
